# A Comparative Analysis of the In Vitro Anticancer Activity of Iridium(III) {η^5^-C_5_Me_4_R} Complexes with Variable R Groups

**DOI:** 10.3390/ijms22147422

**Published:** 2021-07-10

**Authors:** Alice De Palo, Dijana Draca, Maria Grazia Murrali, Stefano Zacchini, Guido Pampaloni, Sanja Mijatovic, Danijela Maksimovic-Ivanic, Fabio Marchetti

**Affiliations:** 1 Department of Chemistry and Industrial Chemistry, University of Pisa, Via Moruzzi 13, I-56124 Pisa, Italy; depaloalice@gmail.com (A.D.P.); mgmurrali@ucla.edu (M.G.M.); guido.pampaloni@unipi.it (G.P.); 2 Department of Immunology, Institute for Biological Research “Sinisa Stankovic”—National Institute of Republic of Serbia, University of Belgrade, 11060 Belgrade, Serbia; dracadiana@gmail.com (D.D.); sanjamama@ibiss.bg.ac.rs (S.M.); 3 Department of Industrial Chemistry “Toso Montanari”, University of Bologna, Viale Risorgimento 4, I-40136 Bologna, Italy; stefano.zacchini@unibo.it

**Keywords:** bioorganometallic chemistry, organoiridium complexes, cytotoxicity, apoptosis, senescence, cell proliferation

## Abstract

Piano-stool iridium complexes based on the pentamethylcyclopentadienyl ligand (Cp*) have been intensively investigated as anticancer drug candidates and hold much promise in this setting. A systematic study aimed at outlining the effect of Cp* mono-derivatization on the antiproliferative activity is presented here. Thus, the dinuclear complexes [Ir(η^5^-C_5_Me_4_R)Cl(μ-Cl)]_2_ (R = Me, **1a**; R = H, **1b**; R = Pr, **1c**; R = 4-C_6_H_4_F, **1d**; R = 4-C_6_H_4_OH, **1e**), their 2-phenylpyridyl mononuclear derivatives [Ir(η^5^-C_5_Me_4_R)(k*_N_*,k*_C_*PhPy)Cl] (**2a–d**), and the dimethylsulfoxide complex [Ir{η^5^-C_5_Me_4_(4-C_6_H_4_OH)}Cl_2_(κ*_S_*-Me_2_S=O)] (**3**) were synthesized, structurally characterized, and assessed for their cytotoxicity towards a panel of six human and rodent cancer cell lines (mouse melanoma, B16; rat glioma, C6; breast adenocarcinoma, MCF-7; colorectal carcinoma, SW620 and HCT116; ovarian carcinoma, A2780) and one primary, human fetal lung fibroblast cell line (MRC5). Complexes **2b** (R = H) and **2d** (4-C_6_H_4_F) emerged as the most active ones and were selected for further investigation. They did not affect the viability of primary mouse peritoneal cells, and their tumoricidal action arises from the combined influence on cellular proliferation, apoptosis and senescence. The latter is triggered by mitochondrial failure and production of reactive oxygen and nitrogen species.

## 1. Introduction

**Figure 1:**
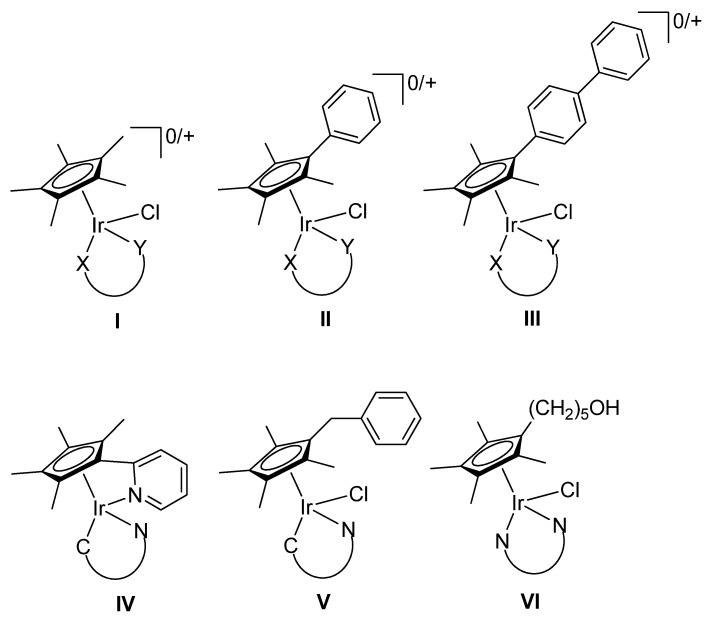
Structures of iridium(III) complexes with penta- and tetramethylcyclopentadienyl ligands and bidentate *N,N*- or 2-arylpyridyl (*C,N*-) ligands, investigated as anticancer drugs. Neutral complexes from anionic *C,N* and *N,N* ligands; monocationic complexes from neutral *N,N* ligands.

Few platinum compounds have been routinely administered in clinical treatments against various types of cancer [1]; however, despite their undoubtful efficacy, they present significant limitations, such as negative side effects, associated phenomena of intrinsic and acquired resistance, a limited number of treatable tumors, and the needing of hospitalization for intravenous administration [2]. These facts have stimulated the research towards new drugs based on different transition metal elements [3]. Indeed, transition metal complexes possess peculiar properties associated with the metal center, which are not available on organic compounds, and thus provide a superior medicinal potential [4]. In this regard, some categories of organometallic complexes have shown a great promise [5], and in particular piano-stool iridium(III) complexes of general formula [IrCp*(X^Y)Cl]^0/+^ (Cp* = η^5^-C_5_Me_5_; X^Y = bidentate neutral ligand or anionic 2-arylpyridyl ligand) have been intensively investigated (Figure 1, structure **I**) [6,7]. Compounds of this type, as well as a variety of their derivatives (see below), are typically accessible from dinuclear precursors upon addition of the bidentate ligand (X^Y) via cleavage of chloride bridges. The presence of the strong donor pentamethylcyclopentadienyl ring provides a stabilizing effect towards the +III oxidation state of the metal, and favors the substitution of the relatively labile chloride ligand. It was found that the replacement of one methyl substituent belonging to Cp* with an aryl group (phenyl or biphenyl, see structures **II** and **III** in Figure 1) generally determines a leap in the antiproliferative activity [8]. Sadler and co-workers attributed this effect to increased lipophilicity and ability to intercalate DNA [9]. Otherwise, the opposite activity trend (i.e., **I** > **II** > **III**) was recently reported for 2-arylpyridyl complexes on A549, HeLa, and BEAS-2B cells by Liu and co-workers, explained on the basis of electronic factors regulating chloride dissociation [10]. Neutral 2-arylpyridyl complexes usually exert a relatively strong cytotoxicity by means of a multimodal action, including alteration of cellular redox balance. Further, significant enhancement of the cytotoxicity is achieved by replacing the chloride ligand with pyridine, which retards hydrolysis and results in a marked propension to trigger ROS production [11].

More recently, Pizarro and co-workers reported that a tether ring structure, accessible via modification of the Cp* with a methylene–pyridine pendant (Figure 1, structure **IV**), exhibits exceptional potency against MCF-7 cells, with respect to analogous benzyl species (structure **V**) which in turn revealed more cytotoxic than the Cp* analogues [12]. Alternative functionalization of Cp* with an alkyl-alcohol group (structure **VI**) has also been evaluated, resulting in modest activity against the A2780 cell line [13].

In this scenario, a comprehensive evaluation of the effects of the modification of the Cp* ring has not been fully addressed hitherto. A recent study by some of us outlined that replacement of one methyl with a range of substituents deeply influences the catalytic activity of the resulting iridium(III) tetramethylcyclopentadienyl complexes in water oxidation, through a balance of electronic and steric factors [14]. Here, we report the synthesis of a series of piano-stool 2-phenylpyridyl complexes containing a {η^5^-C_5_Me_4_R} ligand with variable R, and a study of their in vitro anticancer activity.

## 2. Results and Discussion

### 2.1. Synthesis and Characterization of Compounds

**Scheme 1:**
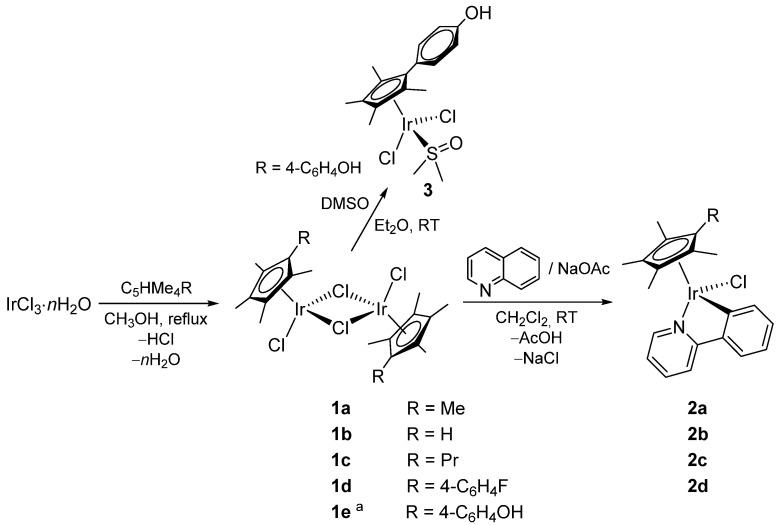
Synthesis of di- and mono-iridium complexes bearing variable tetramethyl-cyclopentadienyl ligands. ^a^ From C_5_HMe_4_(4-C_6_H_4_OCMe_2_OMe).

The neutral di-iridium complexes **1a–e** were obtained from the commercial iridium(III) chloride hydrate by reaction with the appropriate substituted cyclopentadiene precursor in refluxing methanol, following the published procedures (Scheme 1). The synthesis of **1e** required the preliminary protection of the hydroxyl group; thus, the reaction between IrCl_3_·*n*H_2_O and C_5_HMe_4_ (4-C_6_H_4_OCMe_2_OMe) directly afforded **1e** after silica chromatography. All the products showed very low solubilities in water, otherwise **1a–d** were well soluble in chlorinated solvents (i.e., dichloromethane and chloroform), methanol, and dimethylsulfoxide. The hydroxyl function is crucial to solubility; thus, **1e** is slightly soluble in methanol and dimethylsulfoxide but not soluble in chlorinated solvents. The mononuclear 2-phenylpyridyl complexes **2a–d**, including the unprecedented ones **2b–d**, were synthesized by allowing the respective parent compounds **1a–d** to react with a two-fold excess of 2-phenylpyridine in the presence of sodium acetate, in dichloromethane at reflux temperature. After the work-up, **2a–c** were isolated in approximately 75% yields, whereas **2d** was isolated in 40% yield. It was not possible to obtain the 2-phenylpyridyl derivative of **1e**, essentially due to the solubility issues; on the other hand, the mono-iridium dimethylsulfoxide adduct **3** could be easily obtained from **1e** and finally isolated in 71% yield (Scheme 1). The unprecedented mononuclear complexes **2b–d** and **3** are insoluble in water. In contrast to **2b–d**, which are well soluble in dichloromethane and chloroform, **3** manifests a very low solubility even in organic solvents, apart from DMSO. The new compounds were fully characterized by elemental analysis, IR, and NMR spectroscopy (Figures S1–S15). Due to the presence of the chiral iridium center, the NMR spectra of **2b–d** (in CDCl_3_) reveal four non-equivalent methyl groups within the {C_5_Me_4_} moiety; conversely, two resonances for such methyls are recognized in the NMR spectra of **3** (in DMSO-d_6_), this complex lacking asymmetry. The ^19^F NMR resonance due to the fluorine atom in **2d** occurs at −114.5 ppm. The hydroxyl unit in **3** manifests itself with an infrared band at 3262 cm^−1^ and a ^1^H NMR singlet at 9.68 ppm.

**Figure 2:**
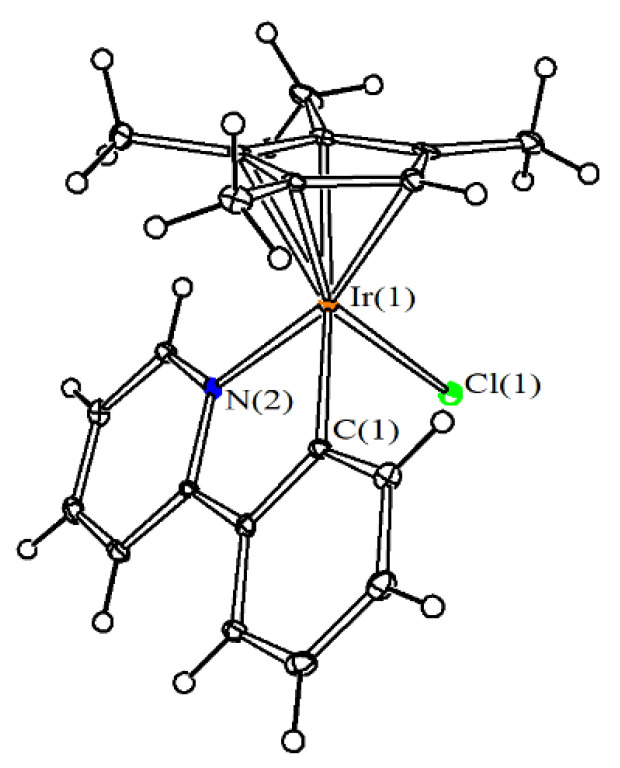
Molecular structure of [Ir(η^5^-C_5_HMe_4_)(κ*_N_*,κ*_C_*PhPy)Cl], **2b**, with key atoms labeled. Displacement ellipsoids are at the 30% probability level. Selected bond distances (Å) and angles (°): Ir(1)−Cl(1) 2.3963(14), Ir(1)−C(1) 2.069(5), Ir(1)−N(2) 2.062(5), Ir(1)−Cp*_average_ 2.196(13), Cl(1)−Ir(1)−C(1) 86.97(15), Cl(1)−Ir(1)−N(2) 86.11(15), C(1)−Ir(1)−N(2) 78.3(2).

**Figure 3:**
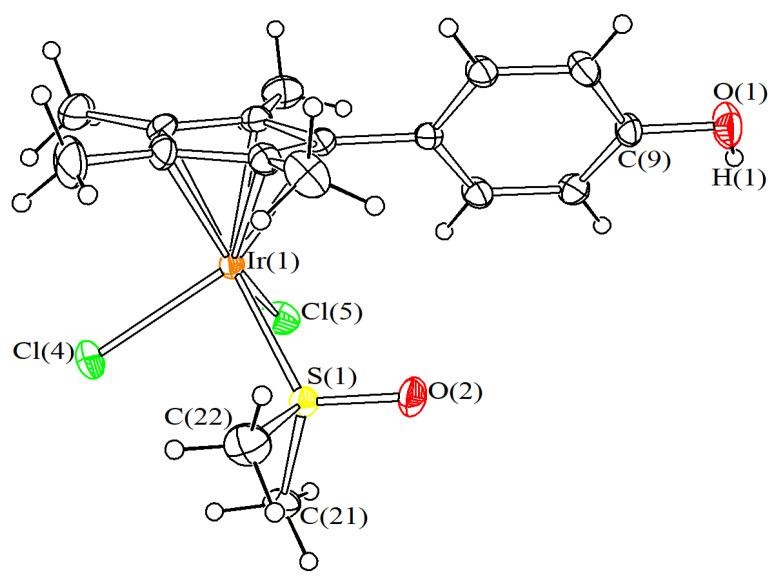
Molecular structure of [Ir{η^5^-C_5_Me_4_(4-C_6_H_4_OH)}Cl_2_(κ*_S_*-Me_2_S=O)], **3**, with key atoms labeled. Displacement ellipsoids are at the 30% probability level. Selected bond distances (Å) and angles (°): Ir(1)−Cl(4) 2.4062(8), Ir(1)−Cl(5) 2.3955(8), Ir(1)−S(1) 2.2995(8), Ir(1)−Cp*_average_ 2.177(7), S(1)-O(2) 1.490(2), S(1)-C(21) 1.779(4), S(1)-C(22) 1.782(4), C(9)-O(1) 1.363(4), Cl(4)−Ir(1)−Cl(5) 89.76(4), Ir(1)-S(1)-O(2) 116.22(11), O(2)-S(1)-C(21) 106.78(16), O(2)-S(1)-C(22) 107.56(19).

The structures of **2b** and **3** were ascertained by single-crystal X-ray diffraction studies. Views of the ORTEP molecular structures are shown in Figure 2 and Figure 3 and details of these studies are provided in Table 1. Both complexes adopt a three-legged piano stool geometry, with bonding parameters comparable to those reported for analogous Ir^III^-complexes containing functionalized Cp ligands and, respectively, additional phenylpyridine [10] and DMSO [15,16,17,18,19] ligands. An inter-molecular H-bond is present in **3**, involving the O(1)-H(1) group and the Me_2_SO ligand [O(1)-H(1) 0.824(19) Å, H(1)**···**O(2)#1 1.99(3) Å, O(1)**···**O(2)#1 2.776(4) Å, <O(1)H(1)O(2)#1 159(5)°; symmetry transformation used to generate equivalent atom #1: −x+1, −y, −z]. The iridium–chlorine bond distance deserves some more comments, in that it follows the order **2a** [2.4046(15) Å] [20] > **2b** [2.3963(14) Å, this work] > [Ir{η^5^-C_5_Me_4_(4-C_6_H_4_Ph)}(κ*_N_*,κ*_C_*PhPy)Cl] [2.3886(8) Å], reflecting the progressive decrease, along the series, of the electron-donor power of the five-membered ligand [21].

Upon dissolution of **2a–d** and **3** into dimethylsulfoxide/water solution (1:3 *v*/*v*), only one set of signals was NMR detected. The NMR patterns did not change after maintaining the solutions at 37 °C for three days. Using dimethylsulfone as an internal standard, it was established that the fraction of residual compound approached 100% in every cases. Analogous evaluation was not reliable for **1b–e**, due to limited solubility of these complexes at 37 °C. The NMR evidence highlights the substantial stability of mono-iridium complexes in the selected aqueous environment, while Sadler previously recognized fast, partial chloride/water exchange from **2a** in CD_3_OD/D_2_O (1:4 *v*/*v*) at room temperature [21]. Overall, these facts suggest that **2a–d** are relatively inert in the presence of water, although sensitive to modifications of the composition of the aqueous medium, and a viable mechanism for their first activation in physiological environment may reasonably involve chloride dissociation. According to UV-Vis spectroscopy, the species derived from dissolution of **2a**, **2c**, and **2d** in RPMI-1640 cell culture medium did not change after being stored at 37 °C for 24 h; conversely, **2b** underwent fast decomposition under these conditions, and the same behavior was recognized for this complex in octanol/water mixture (Log *P_ow_* analysis, vide infra).

### 2.2. Cytotoxicity

The new mono-iridium complexes **2b–d** and **3**, as well as the corresponding precursors **1b–e**, were assessed for their cytotoxic activity (Table 2). The viability of cells was analyzed by two different tests, i.e., MTT (based on mitochondrial respiration) and CV (based on the quantification of adherent, vital cells), with an aim to overcome deficiencies of each method and thus increase the accuracy of the study. In general, IC_50_ values obtained by MTT are significantly lower than those supplied by CV. However, microscopical evaluation of the cells exposed to the treatments showed that the complexes inhibited cell respiration prior affecting cell viability (data not shown), thus rendering CV data more representative of the real viability reduction.

The previously studied Cp* complex **2a** and cisplatin were used as references. The di-iridium complexes **1b** and **1e**, bearing respectively hydrogen and 4-hydroxyphenyl group as R substituents, resulted as substantially inactive, while **1c** and **1d** exhibited a moderate cytotoxicity which is comparable to that of cisplatin only in the case of **1d** against SW620 cells (Figure S16). The mono-iridium derivatives **2b–d** generally showed significantly lower IC_50_ values compared to the corresponding parent compounds **1b–d**. Otherwise, the conversion of **1e** into its dimethylsulfoxide mononuclear adduct is not beneficial to the activity: as a matter of fact, **3** is inactive towards all the investigated cell lines, including those ones on which **1e** exhibits IC_50_ values in the range 80–90 μM. With respect to the reference Cp* compound **2a**, a considerable enhancement of the in vitro anticancer action was recognized with **2d**, in alignment with previous findings highlighting the favorable effect of the introduction of an aryl-moiety on the cyclopentadienyl ring (Table 1, Figure S17). On the other hand, the replacement of one methyl (**2a**) with a longer alkyl chain (propyl, **2c**) does not provide an appreciable effect, except for a slight decrease of activity against SW620 cells and MCF-7. The performance of the tetramethylcyclopentadienyl species **2b** does not significantly differ from that of **2a** in several cell lines, although it is slightly better in B16, SW620, and C6 cell lines, where it approaches **2d**. It does not seem that differences in the antiproliferative activity of **2a–d** are imputable to significantly different degrees of lipophilicity, according to measured octanol-water partition coefficients (Log *P*_ow_, see Experimental). As a matter of fact, the Log *P*_ow_ values of **2a–d** fall in the restricted range 1.3–1.6.

**Figure 4:**
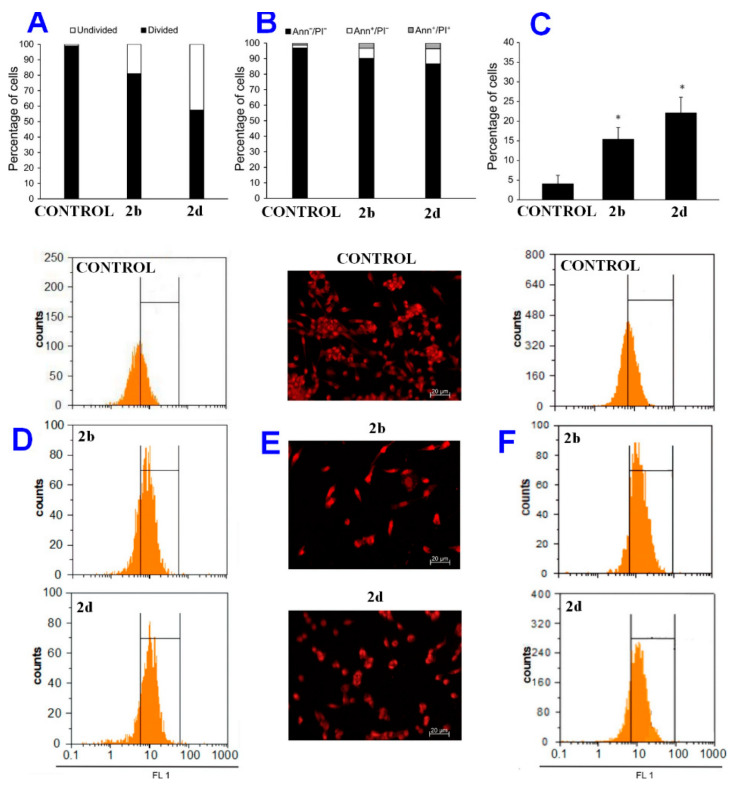
Mechanisms behind A2780 cell viability decrease upon treatment with complexes **2b** and **2d** (see Experimental for details): (**A**) Proliferation potential (CFSE assay); (**B**) apoptosis (AnnV/PI); (**C**) caspase activation (ApoStat assay); (**D**) change in mitochondria membrane potential (DiOC6(3) assay); (**E**) morphology evaluation; (**F**) senescence induction. * Indicates statistically significant values (*p* < 0.05) with respect to the control.

In general, the effect on the viability of human transformed lung fibroblasts, MRC5, is almost the same as on tumor cell lines. The high sensitivity of nonmalignant, primary MRC5 cells toward iridium complexes can be ascribed to the properties of this cell line, resembling the aggressive malignant phenotype, such as high proliferative rate and intracellular features analogous to stem phenotype [22]. Among all tested compounds, **2b** and **2d** were selected as the most promising ones for further studies. Interestingly, both compounds did not affect viability of primary mouse peritoneal cells, thus outlining some tendency to selectivity towards the tumor phenotype (Figure 4).

### 2.3. Mechanism of Action

The analysis of the mechanism of action of **2b** and **2d** revealed inhibition of cell proliferation upon 72 h of incubation (Figure 4A). Inhibited cell division was accompanied by a modest induction of apoptotic cell death for both drugs in caspase dependent manner (Figure 4B,C). The number of cells exhibiting the higher levels of activated caspases was in accordance with the percentage of detected apoptotic cells (see Experimental for details), highlighting the more potent proapoptotic effect of **2d**. Hyperpolarization of mitochondrial membrane preceded mitochondrial collapse and subsequent induction of cell death (Figure 4D) [23]. More in detail, exposure to **2b** and **2d** led to mitochondrial membrane hyperpolarization in the first 24 h of treatment (32.3% and 48.6% in comparison with untreated cells, respectively), suggesting strong influence of iridium complexes on cell respiration and mitochondrial function. Dysfunction of mitochondrial membrane is one of the hallmarks of triggered intrinsic apoptotic pathway, starting as hyperpolarization and finalizing as a loss of membrane potential. Previously, some of us found that a class of *ansa*-titanocene complexes was able to provoke hyperpolarization prior the total mitochondrial collapse [24]. In the present study, staining of cells with propidium iodide permitted to disclose cells with abnormal shape, compressed volume of nuclei, and condensed chromatin, which is typical of apoptotic cells [25], besides cells with giant nuclei, indicating that **2b** and **2d** possess the ability to induce senescence (Figure 4E,F). Cell senescence was further confirmed by increased activity of beta galactosidase, a major senescence biomarker, in cultures exposed to **2b** and **2d** (increase by 38.4 and 31%, respectively, in comparison to the control), Figure 4F. However, a high percentage of autophagosomes was not recognized in treated cultures, indicating irrelevance of autophagic process to the action of iridium complexes (Figures S18–S19). The fact that compounds inhibited cell division and induced cell senescence in parallel with cell death deserves more comments. In fact, while the induction of apoptosis has been reported for diverse iridium complexes, to the best of our knowledge we describe here the first case clearly evidencing the ability of iridium complexes to initiate senescence [26,27]. In recent years, it has been established that senescence is a special form of durable cell cycle arrest, and might represent a primary mechanism for tumor prevention and suppression. There are some examples in the literature demonstrating that the co-existence of senescence and apoptosis can be triggered by the same stressors [28]. To date, it is not clear which of these two alternatives determines the cell destiny, but it is believed that senescence is triggered in cells resistant to apoptosis [28,29,30]. In this regard, several examples are known of cytotoxic drugs able to trigger cell senescence, e.g., cisplatin, bleomycin, and ritonavir [28,29], and much effort is currently directed to develop the so called pro-senescent therapy, as part of differentiation-based therapies. The possibility of inducing cellular senescence and/or differentiation was previously observed for other metal-based drugs such as organotin compounds, either in free form or linked to nano-carriers [31]. The advantage of cell senescence over the killing-based strategies might consist in avoiding apoptosis-induced cell division, which limits the efficacy of chemotherapy [32].

**Figure 5:**
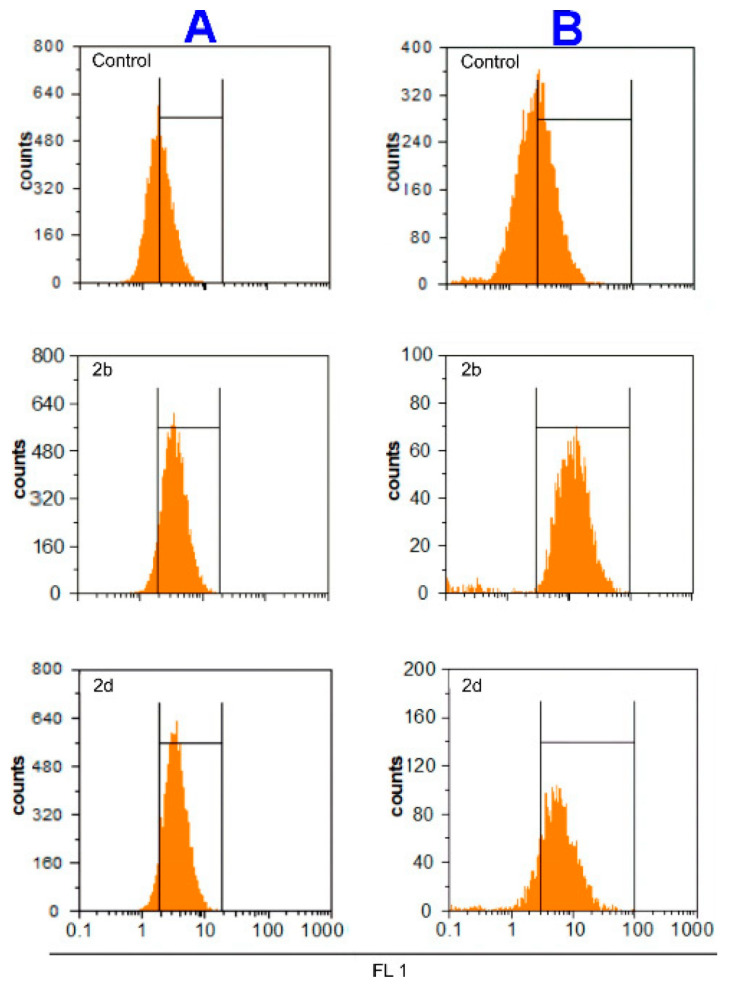
Effect of complexes **2b** and **2d** on the production of reactive nitrogen and oxygen species in A2780 cells: (**A**) nitric oxide (DAF-FM assay); (**B**) hydrogen peroxide, hypochlorous acid, and peroxynitrite (DHR assay). Control: cell culture not treated with the complexes.

For the evaluation of the production of reactive oxygen and nitrogen species (ROS/RNS), two different probes were used. More precisely, the DAF-FM probe detects the intracellular status of nitric oxide, while the DHR123 probe measures the total amount of hydrogen peroxide, hypochlorous acid, and peroxynitrite, accumulated inside the cells during the whole time of incubation. Using the mentioned redox sensitive dyes, a strong increase of intracellular levels of NO (40.9% for **2b** and 38% for **2d**) and of H_2_O_2_, HOCl and ONOO^−^ (in total, 46.4% for **2b** and 36.3% for **2d**) was detected in A2780 cells upon treatment with the complexes, with respect to the untreated cultures (Figure 5). Consistently, Sadler and co-workers observed stimulation of ROS production on analogous complexes. Briefly, they found that iridium(III) cyclopentadienyl complexes with iodide and azopyridine ligands induced ROS burst in human lung cancer cells [11,33]. In addition, related iridium complexes containing a pyridine ligand elicited a high level of ROS production in A2780 cancer cells.

In the present case, the observed affected mitochondrial respiration should be tightly connected to the oxidative stress promoted by the complexes. Having in mind that ROS/RNS species play an essential role in cellular physiology as well as in pathology, from control of cell signaling pathways to induction of death, a fine tuning between proliferation inhibition, apoptosis, and senescence may be of crucial relevance for the activity of a drug candidate.

## 3. Conclusions

Iridium-Cp* complexes constitute a well-established family of organometallic anticancer drug candidates, and previous findings outlined the significant impact of mono-substitution of one Cp*-methyl group on the cytotoxicity. Here, we reported a systematic study aimed at assessing the effect of different substituents. Two series of homologous di- and mononuclear iridium complexes, respectively, were prepared with variable {C_5_Me_4_R} rings, characterized, and assessed for the antiproliferative activity towards a panel of cell lines. The results showed a higher activity for mono-iridium phenyl–pyridyl complexes compared to the respective dinuclear precursors and some effect associated with R. In general, differences in the antiproliferative activity might arise, at least in part, from a different stability of the complexes, leading to a different speciation in the cell environment. While the relatively weak binding of the chloro-bridges in di-iridium compounds is expected to accelerate their disruption, the properties of the R substituent may affect the inertness of the iridium coordination set. Notably, the replacement of one methyl with hydrogen has been proven sufficient to determine fast degradation of the related mononuclear complex in aqueous media; however, this phenomenon leads to enhanced cytotoxicity thus rendering the overall picture not easily rationalizable.

Mononuclear complexes with R = H (**2b**) and 4-C_6_H_4_F (**2d**) exhibited the best performance within the series, displaying IC_50_ values in the low micromolar range, and were thus selected for targeted experiments aimed at elucidating the mode of action. While **2b** and **2d** were inactive towards nontumoral mouse peritoneal cells, they supplied a triple effect on tumor cells, consisting in proliferation inhibition, apoptotic cell death, and senescence induction. Note that induction of senescence has been rarely recognized on organoiridium complexes. The possibility of developing new drugs able to initiate a senescent program is highly desirable, in the light of a convenient mode of action affecting the growth of the malignant cells.

## 4. Experimental

### 4.1. Materials and Methods

Iridium(III) chloride hydrate (99.9%) was purchased from Strem, while organic reactants were purchased from Alfa Aesar, Apollo Sci., and TCI Europe, and were of the highest purity available. Compounds **1a [34]** and **1b–e** [14] were synthesized according to the respective literature procedures. Unless otherwise specified, operations were conducted in air. Silica gel (Merck, 70–230 mesh) was dried at 150 °C overnight. NMR spectra were recorded at 298 K on a Bruker Avance II DRX400 instrument equipped with a BBFO broadband probe. Chemical shifts (expressed in parts per million) are referenced to the residual solvent peaks (^1^H, ^13^C) or to external standard (^19^F, CFCl_3_). ^1^H and ^13^C spectra were assigned with the assistance of DEPT-135 spectra and ^1^H-^13^C (*gs*-HSQC and *gs*-HMBC) correlation experiments [35]. IR spectra of solid samples (650–4000 cm^−1^) were recorded on a Perkin Elmer Spectrum One FT-IR spectrometer, equipped with a UATR sampling accessory. Elemental analyses were performed on a Vario MICRO cube instrument (Elementar).

### 4.2. Synthesis and Characterization of Compounds

**Scheme 2:**
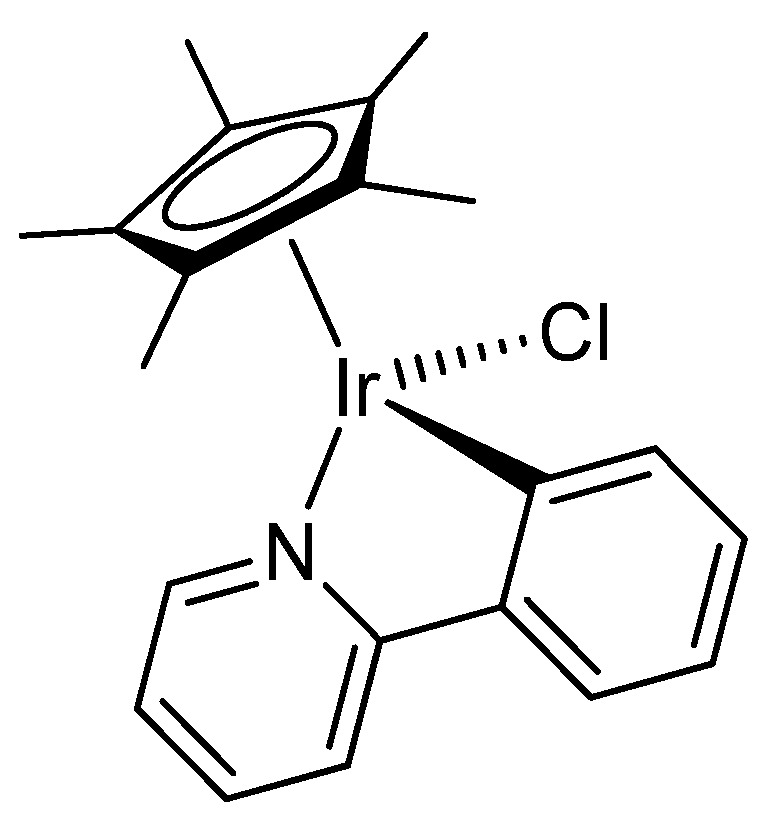
Structure of **2a**.

Synthesis of (1,2,3,4,5-pentamethylcyclopentadienyl)(2-phenylpyridine)iridium(III)chloride, **2a** (Scheme 2).

The title compound was prepared by means of a slightly modified literature procedure [36], which was then applied to the syntheses of **2b–d**. A mixture of [Ir(η^5^-Cp^*^)Cl(µ-Cl)]_2_ (0.100 g, 0.126 mmol), 2-phenylpyridine (37 μL, 0.25 mmol), and sodium acetate (0.041 g, 0.50 mmol) in CH_2_Cl_2_ (15 mL) was refluxed with stirring for 24 h under N_2_ atmosphere. The resulting solution was filtered through celite and the filtrate was evaporated to dryness on a rotary evaporator and then washed with pentane. The obtained suspension was filtered, and the orange solid was dried under vacuum affording **2a**. Yield: 0.093 g, 72%. Analyses calculated for C_21_H_23_ClIrN: C, 48.78; H, 4.48; N, 2.71. Found: C, 48.61; H, 4.54; N, 2.69. IR (solid state): ῦ/cm^−1^ = 3036 w, 2983 w (CH_3_), 2911 w (CH_3_), 1602 s (C = N), 1580 m-s, 1544 w, 1476 s, 1436 m, 1415 s, 1371 m, 1320 m, 1303 w-m, 1268 s, 1228 w-m, 1161 m, 1075 m, 1064 m, 1025 s, 1009 m, 946 w, 890 w, 798 m, 768 vs, 760 vs, 739 vs, 667 m-s. ^1^H NMR (CDCl_3_): δ/ppm = 8.72 (d, ^3^J_HH_ = 5.4 Hz, 1 H, C_5_H_4_N); 7.84 (d, ^3^J_HH_ = 7.6 Hz, 2 H, C_6_H_4_ + C_5_H_4_N); 7.68 (t, ^3^J_HH_ = 7.0 Hz, 2 H, C_6_H_4_ + C_5_H_4_N); 7.23 (t, ^3^J_HH_ = 7.3 Hz, 1 H, C_6_H_4_); 7.12−7.02 (m, 2 H, C_6_H_4_ + C_5_H_4_N); 1.70 (s, 15 H, C_5_*Me*_5_). ^13^C{^1^H} NMR (CDCl_3_): δ/ppm = 167.4, 137.0, 135.8, 131.0, 122.3, 118.9 (C_6_H_4_); 163.4, 151.3, 144.1, 123.9, 122.1 (C_5_H_4_N); 88.5 (*C*_5_Me_5_); 8.9 (C_5_*Me*_5_).

**Scheme 3:**
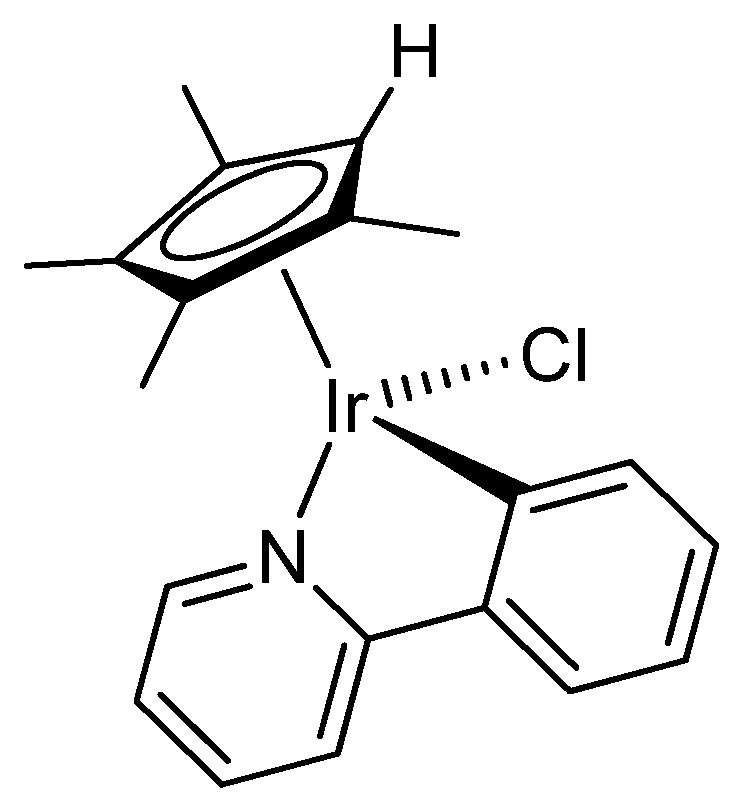
Structure of **2b**.

Synthesis of **t**etramethylcyclopentadienyl)(2-phenylpyridine)iridium(III)chloride, **2b** (Scheme 3).

From **1b** (0.100 g, 0.130 mmol), 2-phenylpyridine (38 μL, 0.26 mmol) and sodium acetate (0.043 g, 0.52 mmol). Orange solid, yield 0.093 g (73%). Analyses calculated for C_20_H_21_ClIrN: C, 47.75; H, 4.21; N, 2.78. Found: C, 47.63; H, 4.26; N, 2.73. IR (solid state): ῦ/cm^−1^ = 2982 vs (CH_3_), 2890s (CH_3_), 1602m (C = N), 1580 m, 1550 w, 1474 s, 1439 m, 1415 m-s, 1380 m-s, 1317 w, 1303 w, 1267 s, 1228 m, 1164 s, 1084 vs-br, 1061 vs, 1024 vs, 957 m-s, 864 w, 799 m-s, 765 vs, 738 vs, 698 m. ^1^H NMR (CDCl_3_): δ/ppm = 8.90 (d, ^3^J_HH_ = 5.5 Hz, 1 H, C_5_H_4_N); 7.90 (d, ^3^J_HH_ = 7.5 Hz, 1 H, C_6_H_4_); 7.83 (d, ^3^J_HH_ = 8.1 Hz, 1 H, C_6_H_4_); 7.69 (dd, ^3^J_HH_ = 14.0, 7.2 Hz, 2 H, C_5_H_4_N); 7.21 (t, ^3^J_HH_ = 7.0 Hz, 1 H, C_6_H_4_); 7.07 (dd, ^3^J_HH_ = 9.0, 6.3 Hz, 2 H, C_5_H_4_N + C_6_H_4_); 4.80 (s, 1H, C_5_Me_4_*H*); 1.76 (d, ^3^J_HH_ = 1.9 Hz, 6 H, C_4_*Me*_4_); 1.71, 1.67 (s, 6 H, C_4_*Me*_4_). ^13^C{^1^H} NMR (CDCl_3_): δ/ppm = 167.3, 144.3, 136.5, 131.0, 122.4, 119.0 (C_6_H_4_); 161.9, 153.2, 137.1, 124.1, 122.2 (C_5_H_4_N); 96.9, 91.2, 90.6, 84.5 (*C*_4_Me_4_); 75.2 (CH); 10.9, 10.5, 8.8, 8.6 (C_4_*Me*_4_). Crystals of **2b** suitable for X-ray analysis were obtained by slow evaporation of the solvent from hexane solution.

**Scheme 4:**
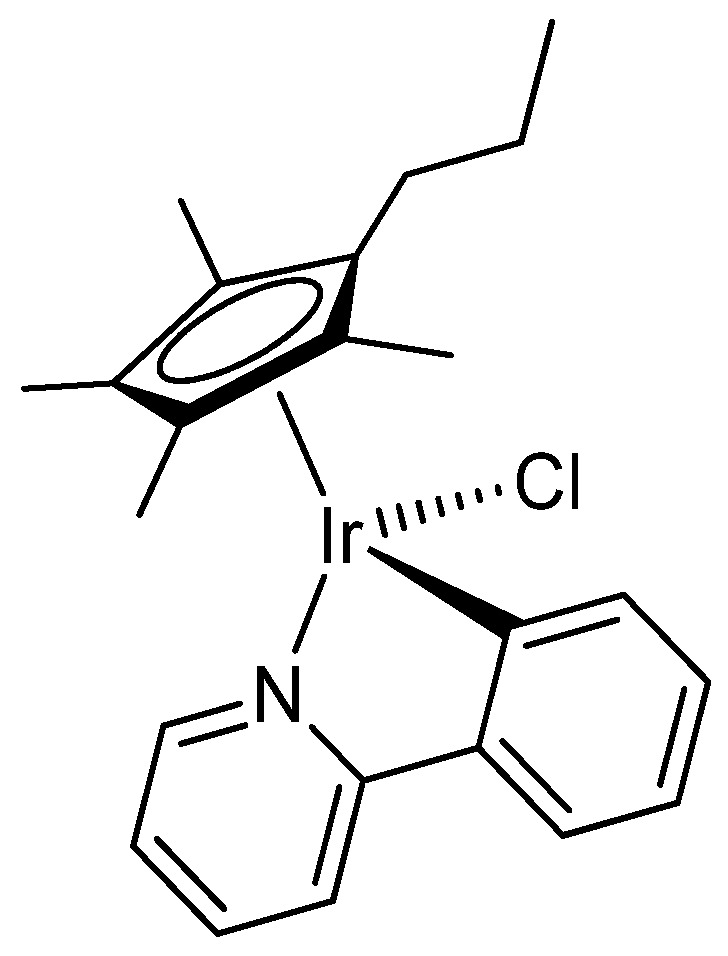
Structure of **2c**.

Synthesis of (1-(propyl)-2,3,4,5-tetramethylcyclopentadienyl)(2-phenylpyridine)iridium(III)chloride, **2c** (Scheme 4).

From **1c** (0.100 g, 0.117 mmol), 2-phenylpyridine (34 μL, 0.23 mmol) and sodium acetate (0.038 g, 0.47 mmol). Orange solid, yield 0.093 g (75%). Analyses calculated for C_23_H_27_ClIrN: C, 50.67; H, 4.99; N, 2.57. Found: C, 50.74; H, 4.91; N, 2.63. IR (solid state): ῦ/cm^−1^ = 3058 w, 2961 w (C*sp_3_*H), 2932 w-m (C*sp_3_*H), 2859 w (C*sp_3_*H), 1602 s (C = N), 1580 s, 1561 w-m, 1549 w-m, 1498 w, 1474 vs, 1455 vs, 1437 s, 1413 s, 1373 m-s, 1338 w, 1317 m-s, 1302 m-s, 1266 s, 1242 w, 1226 w, 1163 m-s, 1123 w, 1109 w, 1084 w, 1060 m, 1027 vs, 982 w, 861 w, 815 w, 798 m, 757 vs, 733 vs, 702 w, 668 w, 657 w. ^1^H NMR (CDCl_3_): δ/ppm = 8.72 (d, ^3^J_HH_ = 5.4 Hz, 1 H, C_5_H_4_N); 7.83 (dd, ^3^J_HH_ = 12.1, 8.0 Hz, 2 H, C_6_H_4_); 7.67 (dd, ^3^J_HH_ = 14.4, 7.5 Hz, 2 H, C_5_H_4_N); 7.22 (t, ^3^J_HH_ = 7.3 Hz, 1 H, C_6_H_4_); 7.07 (dt, ^3^J_HH_ = 14.1, 7.0 Hz, 2 H, C_6_H_4_ + C_5_H_4_N); 2.13−2.02 (m, 2 H, CH_2_); 1.74, 1.72, 1.71, 1.69 (s, 12 H, C_4_*Me*_4_); 1.49 (dd, ^3^J_HH_ = 15.0, 7.5 Hz, 2 H, C*H*_2_CH_3_); 0.97 (t, ^3^J_HH_ = 7.3 Hz, 3 H, CH_2_C*H*_3_). ^13^C{^1^H} NMR (CDCl_3_): δ/ppm = 167.4, 137.1, 136.0, 131.0, 122.4, 118.9 (C_6_H_4_); 163.3, 151.6, 144.2, 123.9, 122.1 (C_5_H_4_N); 90.5, 89.9, 89.0, 88.7 (*C*_4_Me_4_); 88.2 (*C*-Pr); 26.2 (CH_2_); 22.3 (*C*H_2_CH_3_); 14.4 (CH_2_*C*H_3_); 9.1, 9.0 (C_4_*Me*_4_).

**Scheme 5:**
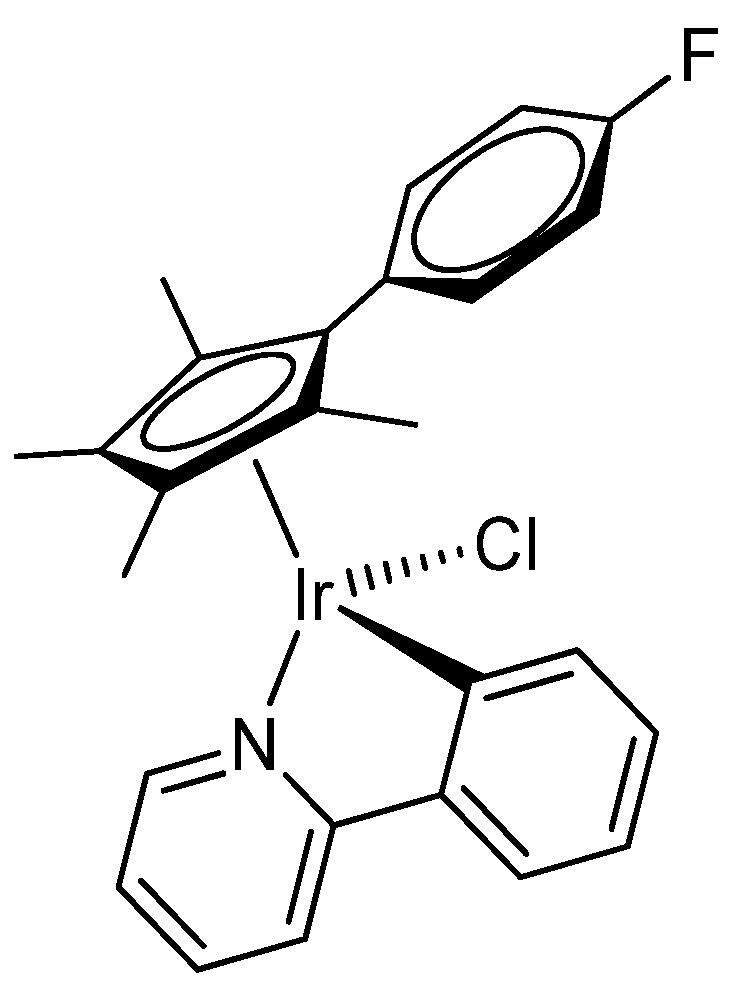
Structure of **2d**.

Synthesis of (1-(4-fluorobenzene)-2,3,4,5-tetramethylcyclopentadienyl)(2-phenylpyridine)iridium(III)chloride, **2d** (Scheme 5).

From **1d** (0.114 g, 0.119 mmol), 2-phenylpyridine (35 μL, 0.24 mmol) and sodium acetate (0.039 g, 0.48 mmol). Orange solid, yield 0.053 g (40%). Analyses calculated. for C_26_H_24_ClFIrN: C, 52.30; H, 4.05; N, 2.35. Found: C, 52.24; H, 4.11; N, 2.29. IR (solid state): ῦ/cm^−1^ = 2982 vs (CH_3_), 2890 m (CH_3_), 1604 m (C = N), 1582 w-m, 1562 w, 1519 s, 1476 s, 1416 m, 1383 s, 1317 w, 1303w, 1267 m, 1226 s, 1158 vs, 1093 s, 1028 m-s, 954 m-s, 845 m-s, 818 s, 796 m, 756 vs, 736 s. ^1^H NMR (CDCl_3_): δ/ppm = 8.51 (d, ^3^J_HH_ = 5.5 Hz, 1 H, C_5_H_4_N); 7.84 (d, ^3^J_HH_ = 8.0 Hz, 1 H, C_6_H_4_); 7.71 (d, ^3^J_HH_ = 7.6 Hz, 1 H, C_6_H_4_); 7.67 (d, ^3^J_HH_ = 7.7 Hz, 2 H, C_6_H_4_ + C_5_H_4_N); 7.49−7.43 (m, 2 H, C_6_H_4_); 7.17 (t, ^3^J_HH_ = 7.2 Hz, 1 H, C_6_H_4_); 7.10 (d, ^3^J_HH_ = 8.7 Hz, 2 H, C_6_H_4_); 7.07 (d, ^3^J_HH_ = 7.8 Hz, 1 H, C_5_H_4_N); 6.99 (t, ^3^J_HH_ = 6.3 Hz, 1 H, C_5_H_4_N); 1.83, 1.76, 1.73, 1.59 (s, 12 H, C_4_*Me*_4_). ^13^C{^1^H} NMR (CDCl_3_): δ/ppm = 167.4, 137.2, 131.1, 124.0, 122.5, 119.0 (C_6_H_4_); 162.2, 151.3, 144.0, 135.6, 122.4 (C_5_H_4_N); 162.1 (d, ^2^J_CF_ = 247 Hz, CF); 132.7, 132.6, 128.5, 115.9, 115.6 (C_6_H_4_); 99.1, 97.7, 86.4, 84.2, 83.1 (C_5_); 10.0, 9.7, 8.8 (C_4_*Me*_4_). ^19^F{^1^H} NMR (CDCl_3_): δ/ppm = −114.5.

**Scheme 6:**
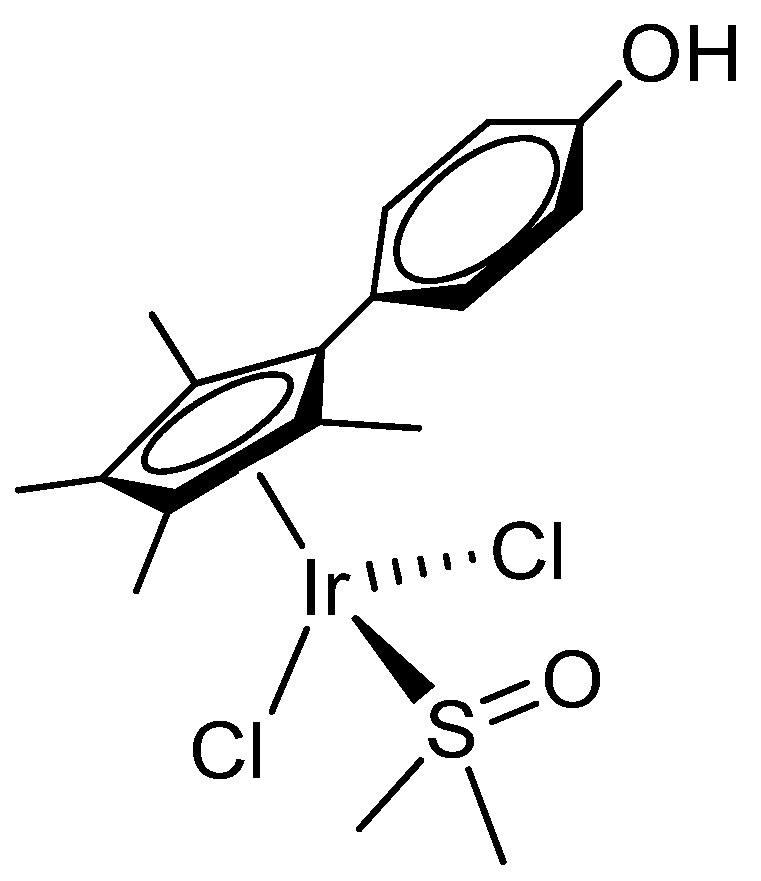
Structure of **3**.

Synthesis of (4-(2,3,4,5-tetramethylcyclopenta-1,4-dien-1-yl)phenol)dimethylsulfoxide iridium(III)dichloride, **3** (Scheme 6).

A suspension of **1e** (150 mg, 0.157 mmol) in Et_2_O (40 mL) was treated with dimethylsulfoxide (0.2 mL), and the resulting mixture was stirred for 18 h. A red-orange microcrystalline solid was obtained, which was isolated and dried under vacuum. Yield 123 mg, 71%. Analyses calculated for C_17_H_23_Cl_2_IrO_2_S: C, 36.82; H, 4.18; S, 5.78. Found: C, 36.65; H, 4.16; S, 5.69. IR (solid state): ῦ/cm^−1^ = 3262 w-m (OH), 2963 w (CH_3_), 2916 w (CH_3_), 2265 w, 1615 m (C = N), 1591 m, 1523 s, 1444 m, 1378 m, 1362 w, 1272 s, 1233 s, 1207 w, 1175 w-m, 1108 m (SO), 1085 vs, 1026 s, 1004 s, 853 s, 817 vs, 771 m, 658 m. ^1^H NMR (DMSO-d_6_): δ/ppm = 9.68 (s, 1 H, OH); 7.39, 6.76 (d, ^3^J_HH_ = 7.8 Hz, 4 H, C_6_H_4_); 1.69, 1.65 (s, 12 H, C*Me*). ^13^C{^1^H} NMR (DMSO-d_6_): δ/ppm = 157.9 (C-OH); 132.1, 115.6 (C_6_H_4_); 120.0 (C_5_-*C*); 100.7, 90.0 (*C*_5_Me_4_); 87.7 (*C*-C_6_H_4_); 10.0, 8.8 (C_4_*Me*_4_). Me_2_SO not observed. Crystals of **3** suitable for X-ray analysis were collected by slow diffusion of hexane into a dichloromethane solution stored at −30 °C.

### 4.3. X-ray Crystallography

Crystal data and collection details for **2b** and **3** are reported in Table 1. Data were recorded on a Bruker APEX II diffractometer equipped with PHOTON2 (**2b**) or CCD (**3**) detector using Mo–Kα radiation. Data were corrected for Lorentz polarization and absorption effects (empirical absorption correction SADABS) [37]. The structures were solved by direct methods and refined by full-matrix least-squares based on all data using *F*^2^ [38]. Hydrogen atoms were fixed at calculated positions and refined by a riding model. All non-hydrogen atoms were refined with anisotropic displacement parameters.

### 4.4. Behavior in Aqueous Solutions

(**a**) Octanol/water partition coefficients (Log P_ow_). Partition coefficients (P_ow_; IUPAC: K_D_ partition constant [39]), defined as P_ow_ = c_org_/c_aq_, where c_org_ and c_aq_ are molar concentrations of the selected compound in the organic and aqueous phase, respectively, were determined by the shake-flask method and UV-Vis measurements [40]. Deionized water and 1-octanol were vigorously stirred for 24 h, to enable saturation of both phases, then separated by centrifugation. A stock solution of the selected iridium compound (ca. 2 mg) was prepared by first adding acetone (50 μL, to help solubilization), followed by water-saturated octanol (2.5 mL). The solution was diluted with water-saturated octanol (ca. 1:3 *v*/*v* ratio, c_Ir_ ≈ 10^−4^ M, so that 1.5 ≤ A ≤ 2.0 at λ_max_) and its UV-Vis spectrum was recorded (A^0^_org_). An aliquot of the solution (V_org_ = 1.2 mL) was transferred into a test tube and octanol-saturated water (V_org_ = V_aq_ = 1.2 mL) was added. The mixture was vigorously stirred for 30 min at 21 °C then centrifuged (5000 rpm, 5 min). The UV-Vis spectrum of the organic phase was recorded (A^f^_org_) and the partition coefficient was calculated as P_ow_ = A^f^_org_/(A^0^_org_ − A^f^_org_) where A^0^_org_ and A^f^_org_ are the absorbance in the organic phase before and after partition with the aqueous phase, respectively. The wavelength of the maximum absorption of each compound (ca. 300 nm) was used for UV-Vis quantitation. The procedure was repeated three times for each sample (from the same stock solution); results are given as mean ± standard deviation. Log P_ow_ values were as follows: **2a**: 1.47 ± 0.07; **2b**: n/a (extensive degradation of the complex occurred during analysis); **2c**: 1.5 ± 0.2; **2d**: 1.3 ± 0.2. Literature value for **2a**: 1.57 ± 0.08 [41].

(**b**) Stability in D_2_O

Complexes were dissolved in acetone-d_6_/D_2_O 2:1 (**1b,d**), DMSO-d_6_/D_2_O 2:1 (**2a–d** and **3**), or methanol-d_4_/D_2_O 2:1 (**1c**) ([Ir] ≈ 10^−3^ mol L^−1^), then dimethyl sulfone (0.05 mL, 0.164 mmol) was added as internal standard. The mixtures were filtered through a celite pad, and the filtrated solutions were analyzed by ^1^H NMR spectroscopy. NMR data from **2a–d** and **3** are reported in the following (Figures S10–S15). Line broadening observed for **2a** and **3** is probably consequence of the low solubility of these complexes in the employed medium.

**2a**. ^1^H NMR (DMSO-d_6_/D_2_O): δ/ppm = 8.66 (br, 1 H, C_5_H_4_N); 8.18 (br, 1 H, C_6_H_4_); 8.05 (br, 1 H, C_6_H_4_); 7.90 (br, 1 H, C_5_H_4_N); 7.62 (br, 1 H, C_5_H_4_N); 7.43, 7.29 (br, 3 H, C_5_H_4_N + C_6_H_4_); 1.62 (s, 15 H, Me).

**2b**. ^1^H NMR (DMSO-d_6_/D_2_O): δ/ppm = 8.84 (d, J = 4.9 Hz, 1 H, C_5_H_4_N); 8.17 (d, J = 8.3 Hz, 1 H, C_6_H_4_); 8.04 (m, 1 H, C_6_H_4_); 7.90 (m, 1 H, C_5_H_4_N); 7.69 (d, J = 6.7 Hz, 1 H, C_5_H_4_N); 7.39, 7.28 (m, 3 H, C_5_H_4_N + C_6_H_4_); 5.49 (s, 1 H, C_5_Me_4_*H*); 1.74, 1.65 (s, 6 H, Me); 1.54 (s, 6 H, Me).

**2c**. ^1^H NMR (DMSO-d_6_/D_2_O): δ/ppm = 8.66 (d, J = 5.4 Hz, 1 H, C_5_H_4_N); 8.17 (d, J = 7.8 Hz, 1 H, C_6_H_4_); 8.05 (m, 1 H, C_6_H_4_); 7.90 (d, J = 6.9 Hz, 1 H, C_5_H_4_N); 7.63 (t, 1 H, C_5_H_4_N); 7.42 (t, 1 H, C_6_H_4_); 7.28 (m, 3 H, C_6_H_4_ + C_5_H_4_N); 1.96, 1.37 (m, 4 H, CH_2_); 1.64 (s, 12 H, C_4_*Me*_4_); 0.82 (t, ^3^J_HH_ = 7.3 Hz, 3 H, CH_2_C*H*_3_).

**2d**. ^1^H NMR (DMSO-d_6_/D_2_O): δ/ppm = 8.44 (d, J = 5.4 Hz, 1 H, C_5_H_4_N); 8.21 (d, J = 7.8 Hz, 1 H, C_6_H_4_); 8.01 (m, 1 H, C_6_H_4_); 7.95, 7.59 (m, 2 H, C_6_H_4_ + C_5_H_4_N); 7.29 (m, 3 H, C_6_H_4_); 7.05 (m, 4 H, C_6_H_4_ + C_5_H_4_N); 1.95, 1.81, 1.71, 1.47 (s, 12 H, Me). ^19^F{^1^H} NMR (DMSO-d_6_/D_2_O): δ/ppm = 112.5.

**3**. ^1^H NMR (DMSO-d_6_/D_2_O): δ/ppm = 7.35, 6.76 (br, 4 H, arom CH); 1.64, 1.68 (s-br, 12 H, C_5_Me_4_).

The solutions were then stored for 72 h at 37 °C, and new NMR analyses were subsequently performed. Integral ratios referred to the standard, measured respectively after and before heating, allowed us to estimate the fraction of unaltered compound: **2a**, 98%; **2b**, 98%; **2c**, 98%; **2d**, 97%; **3**, 98%. In the cases of **1b**, **1c**, and **1d**, progressive precipitation of a solid was observed from the solutions over 72 h, presumably due to limited solubility of the compounds; however, no significant variations were detected in the ^1^H NMR spectra. Complex **1e** could not be NMR analyzed due to insufficient solubility in aqueous media, except in the presence of DMSO where, however, **1e** undergoes rapid conversion into **3**. 

(**c**) Stability in cell culture medium

Complexes **2a–d** (ca. 2 mg) were dissolved in the minimum volume of methanol, then the solutions were diluted with RPMI-1640 cell culture medium up to ca. 10^−6^ M [Ir] concentration. UV-Vis spectra were recorded immediately after the preparation of these solutions, showing the maximum absorption at ca. 300 nm except in the case of **2b**. The solutions were stored at 37 °C for 24 h, then new spectra were recorded without displaying significant changes.

### 4.5. Biological Studies

#### 4.5.1. Reagents and Cells

Fetal bovine serum (FBS), RPMI-1640, phosphate-buffered saline (PBS), dimethyl sulfoxide (DMSO), 3-(4,5-dimethylthiazol-2-yl)-2,5-diphenyltetrazolium bromide (MTT), carboxyfluorescein diacetate succinimidyl ester (CFSE), crystal violet (CV), fluorescein di(β-D-galactopyranoside) (FDG), dimethylformamide (DMF), propidium iodide (PI), and cisplatin (cPt) were purchased from Merck (St. Louis, MO, USA). Paraformaldehyde (PFA) was purchased from Serva (Heidelberg, Germany), penicillin/streptomycin solution from Biological Industries (Cromwell, CT, USA), acridine orange (AO) from Labo-Moderna (Paris, France), annexin V-FITC (AnnV) from Santa Cruz Biotechnology (Dallas, TX, USA), ApoStat was from R&D Systems (Minneapolis, MN, USA), 3,3′-Dihexyloxacarbocyanine Iodide (DiOC6(3)) and 4-Amino-5-Methylamino-2′,7′-Difluorofluorescein Diacetate (DAF-FM Diacetate) from Molecular Probes (Eugene, OR, USA), and dihydrorhodamine 123 (DHR) from Thermo Fisher Scientific (Waltham, MA, USA). The rat astrocytoma C6 cell line and murine melanoma B16 cell line were kindly gifted by Dr. L. Harhaji–Trajkovic, Institute for Biological Research “Sinisa Stankovic”—National Institute of Republic of Serbia (IBISS), and from Dr. Sinisa Radulovic (Institute for Oncology and Radiology of Serbia), respectively. Human colorectal (SW620 and HCT116), breast (MCF-7), and ovarian (A2780) cancer cell lines and non-malignant fibroblast cell line (MRC5) were purchased from the American Type Culture Collection (ATCC, Manassas, Virginia, USA). Cells were grown in HEPES-buffered RPMI-1640 medium supplemented with 10% FBS, 2 mM L-glutamine, 0.01% sodium pyruvate, and antibiotics (penicillin 100 units/mL and streptomycin 100 μg/mL) at 37 ºC in a humidified atmosphere with 5% CO_2_. For viability assays, cells were seeded in listed densities/well: B16 and C6: 3 × 10^3^; SW620: 9 × 10^3^; A2780 and HCT116: 5 × 10^3^; MCF-7: 8 × 10^3^ and MRC5: 7 × 10^3^. For flow cytometry, A2780 cells were seeded at 2 × 10^5^ density/well in 6-well plates. Peritoneal macrophages were collected from the peritoneal cavity of C57BL/6 mice from the animal facility of IBISS by cavity lavage with ice-cold PBS. Cells were grown in HEPES-buffered RPMI-1640 medium supplemented with 5% FCS, 2 mM L-glutamine, 0.01% sodium pyruvate, penicillin (100 units/mL), and streptomycin (100 μg/mL) at 37 °C in a humidified atmosphere with 5% CO_2_. Macrophages were counted, seeded at 2 × 10^5^ cells/well in 96-well plates for viability assay, and then left for 2 h to adhere. Before treatment, non-adherent cells were removed. The handling of animals and protocol for obtaining macrophages is in the agreement with the rules of the European Union and approved by the Institutional Animal Care and Use Committee at IBISS (No. 02-09/16).

#### 4.5.2. Preparation of Drug Solutions

DMSO stock solutions of **2a**, **2b**, **2c**, **2d**, and **3** were prepared at a concentration of 100 mM and kept at −20 °C. Stock solutions of **1b**, **1c**, **1d**, and **1e** were prepared in ethanol at concentration of 20 mM and kept at −20 °C, while 10 mM stock solution of cisplatin was prepared in DMF just before the usage. Desired final concentrations were obtained by dilution in culture medium. 

#### 4.5.3. Colorimetric Assays for Cellular Viability

Cells were treated with various concentrations (up to 50 or 100 µM) of the above-mentioned complexes for 72 h. For the detection of mitochondrial respiration, cells were cultivated in the presence of MTT staining solution (0.5 mg/mL) for approximately 1 h at 37 °C. The dye was then discarded, and the formed formazan crystals (purple) were dissolved in DMSO. For an evaluation of viable attached cells, cells were fixed with 4% PFA for 10 min at room temperature, and subsequently stained for 15 min with 0.02% CV solution. Cells were then washed with tap water, dried in air, and the CV dye was dissolved in 33% acetic acid. The absorbance was measured with an automated microplate reader at 540 nm, with a reference wavelength of 670 nm. The IC_50_ values were calculated using a four-parameter logistic function and presented as mean ± SD from three independent experiments. 

#### 4.5.4. Flow Cytometry 

For better insight into the mechanisms of action of complexes **2b** and **2d** on A2780 cell line, cells were incubated with **2b** and **2d** (IC_50_ doses) and analyzed by flow cytometry. Several staining protocols were performed: (a) AnnV/propidium iodide (PI) for the detection of apoptotic cell death; (b) ApoStat for detection of caspase activity; (c) AO for the detection of autophagosomes; (d) CFSE for monitoring the influence on cellular proliferation; (e) DAF-FM diacetate for detection of intracellular nitric oxide (NO); (f) DHR for the detection of reactive oxygen/nitrogen species (ROS/RNS); (g) FDG assay for detecting senescence; and h) DiOC6(3) to measure mitochondrial membrane potential. Results were obtained with CyFlow^®^ Space Partec using the PartecFloMax^®^ software. Experiments were carried out in three independent replicates. For AnnV/PI, AO and ApoStat staining, cells were treated with **2b** and **2d** during 48 h, and then detached and washed with PBS. Afterwards, cells were stained, in accordance with the manufacturer’s protocols, with AnnV/PI (15 min, room temperature) in AnnV-binding buffer, or ApoStat (30 min, 37 °C) in PBS 5% FBS, or AO (100 μM; 15 min, 37 °C), in PBS. Finally, cells were washed, resuspended in PBS (or in AnnV-binding buffer for AnnV/PI), and analyzed. For CFSE staining, cells were pre-stained with a PBS solution of CFSE (1 μM; 10 min, 37 °C), washed, seeded, and then exposed to **2b** and **2d** for 72 h. Cells were then washed, trypsinized, dissolved in PBS, and analyzed. Similarly to CFSE, DHR staining was performed with cells being pre-stained first, with 1 μM DHR for 20 min at 37 °C, and then exposed to the experimental compounds for 48 h. At the end of cultivation, cells were washed, detached, and analyzed. For DAF-FM staining, cells were exposed the experimental compounds for 48 h, washed with PBS, trypsinized, and stained with 5 μM of DAF-FM diacetate for 1 h at 37 °C in phenol red-free RPMI 1640. Thereafter, cells were washed and additionally incubated for 15 min in fresh RPMI 1640 without phenol red and serum, to finish the reaction of de-esterification. For the FDG assay, cells were treated with **2b** and **2d** during 48 h, and then stained with β-galactosidase substrate FDG to a final concentration of 1 mM. After 1 min incubation at 37 °C, cells were analyzed. DiOC6(3) staining protocol considered the exposure of treated cells (**2b** and **2d** during 24 h) to 70 nM of DiOC6(3) in PBS during 20 min at 37 °C. Channels FL1 (green emission), FL2 (orange emission), and/or FL3 (dark red emission) were used for fluorescence detection, according to the specific staining agent.

#### 4.5.5. Fluorescence Microscopy

Cells were cultivated on chamber slides overnight (4 × 10^4^/well), then treated with **2b** and **2d** during 48 h. Cells were washed with PBS, fixed with 4% PFA during 15 min at room temperature, and stained with a solution of propidium iodide (50 μg/mL) with 0.1% Triton X-100, 0.1 mM EDTA pH 8.0, and RNase (85 μg/mL) in PBS for 2 min. Finally, cells were washed with PBS and prepared for fluorescence microscopy by layering with fluorescent mounting medium (Dako, Glostrup, Denmark). The slides were analyzed with a Zeiss AxioObserver Z1 inverted fluorescence microscope (Carl Zeiss AG, Oberkochen, Germany) at 200× magnification.

#### 4.5.6. Statistical Analysis

Analysis of variance (ANOVA) followed with a Student–Newman–Keuls test was used for significance of the differences between treatments, and a *p*-value less than 0.05 was taken as statistically significant.

## Figures and Tables

**Table 1:** Crystal data and measurement details for **2b** and **3**.

	2b	3
Formula	C_20_H_21_ClIrN	C_17_H_23_Cl_2_IrO_2_S
FW	503.03	554.51
T, K	100(2)	293(2)
*λ*, Å	0.71073	0.71073
Crystal system	Monoclinic	Monoclinic
Space group	*P*2_1_/*n*	*P*2_1_/*c*
*a*, Å	14.4923(5)	13.9835(5)
*b,* Å	7.3769(3)	8.8692(3)
*c*, Å	15.5178(5)	15.3611(6)
*β*,°	95.9050(10)	96.317(2)
Cell volume, Å^3^	1650.18(10)	1893.55(12)
Z	4	4
*D_c_*, g∙cm^−3^	2.025	1.945
μ,mm^−1^	8.251	7.449
F(000)	968	1072
Crystal size, mm	0.16 × 0.13 × 0.10	0.18 × 0.13 × 0.10
θ limits,°	1.831–25.999	1.465–25.992
Reflections collected	21,455	28,963
Independent reflections	3237 [*R*_int_ = 0.0255]	3715 [*R*_int_ = 0.0394]
Data/restraints/parameters	3237/36/213	3715/1/217
Goodness on fit on F^2^	1.390	1.068
*R*_1_ (*I* > 2σ(*I*))	0.0275	0.0193
*wR*_2_ (all data)	0.0702	0.0487
Largest diff. peak and hole, e Å^−3^	1.648/–2.034	0.497/–1.017

**Table 2:** IC_50_ values (μM) determined for iridium complexes and cisplatin (columns) on cell lines (rows) after 72 h exposure. Values are given as the mean ± SD. Green: MTT assay; yellow: CV assay. B16: mouse melanoma; SW620: human colorectal adenocarcinoma; C6: rat glioma; MCF-7: human breast cancer; HCT116: human colorectal carcinoma; A2780: human ovarian cancer; MRC5: human fibroblast.

	1b	1c	1d	1e	2a	2b	2c	2d	3	Cisplatin
B16	>50	28 ± 4	20.4 ± 0.1	>100	5.1 ± 0.1	2.5 ± 0.2	6.7 ± 0.6	1.2 ± 0.2	>100	5.6 ± 0.2
B16	>50	44 ± 8	28.1 ± 0.1	>100	6 ± 2	3.0 ± 0.1	8 ± 2	1.7 ± 0.4	>100	11.6 ± 0.3
SW620	>50	27 ± 2	8 ± 2	>100	7.4 ± 0.9	2.6 ± 0.1	10.4 ± 0.4	2.0 ± 0.1	>100	11.6 ± 0.1
SW620	>50	45 ± 8	18.5 ± 0.4	>100	5.3 ± 0.2	2.9 ± 0.5	12.4 ± 1.5	2.0 ± 0.2	>100	11 ± 3
C6	>50	27 ± 3	34 ± 2	90 ± 14	4.9 ± 0.8	2.4 ± 0.3	4.0 ± 0.3	2.00 ± 0.01	>100	0.6 ± 0.2
C6	>50	54 ± 5	49 ± 1	>100	4.7 ± 0.5	2.6 ± 0.1	5.9 ± 0.5	2.1 ± 0.1	>100	1.2 ± 0.1
MCF-7	>50	28 ± 5	9.2 ± 0.5	88 ± 12	15 ± 5	8.6 ± 0.2	12.0 ± 1.3	2.5 ± 0.2	>100	0.8 ± 0.2
MCF-7	>50	55 ± 8	30.0 ± 0.4	94 ± 8	13.3 ± 0.6	37 ± 3	17.2 ± 0.1	6.8 ± 0.7	>100	1.2 ± 0.3
HCT116	>50	30 ± 4	8.1 ± 0.7	80 ± 3	4.8 ± 0.9	8.0 ± 0.2	8.0 ± 0.6	1.3 ± 0.3	>100	1.5 ± 0.1
HCT116	>50	38 ± 3	14.5 ± 1.5	79 ± 8	24.6 ± 0.5	7 ± 2	13.0 ± 1.1	5.3 ± 1.3	>100	8.0 ± 0.7
A2780	>50	13 ± 1	16.0 ± 0.3	N.A.	2.9 ± 0.1	5.5 ± 0.5	7.3 ± 0.6	1.3 ± 0.1	N.A.	1.9 ± 0.2
A2780	>50	39 ± 7	20.2 ± 0.3	N.A.	12.3 ± 0.3	4.4 ± 0.6	7.2 ± 0.8	1.8 ± 0.1	N.A.	3.8 ± 0.5
MRC5	>50	36 ± 2	19.1 ± 0.2	>100	2.5 ± 0.5	6.7 ± 0.2	3.7 ± 0.2	2.4 ± 0.3	>100	0.6 ± 0.2
MRC5	>50	>50	22.2 ± 0.1	>100	5.8 ± 0.1	6 ± 2	2.7 ± 0.1	2.1 ± 0.6	>100	0.8 ± 0.1

## Data Availability

The data presented in this study are available on request from the corresponding authors.

## Supplementary Materials

NMR spectra of complexes; details of biological studies. CCDC reference numbers 2086825 (**2b**) and 2086824 (**3**) contain the supplementary crystallographic data for the X-ray studies reported in this paper. These data can be obtained free of charge at www.ccdc.cam.ac.uk/conts/retrieving.html accessed on 4 June 2021 (or from the Cambridge Crystallographic Data Centre, 12, Union Road, Cambridge CB2 1EZ, UK; e-mail: deposit@ccdc.cam.ac.uk).

## Webpage

https://people.unipi.it/fabio_marchetti1974/, accessed on 4 June 2021.
