# Supplementary material for: A Comparative Analysis of the In Vitro Anticancer Activity of Iridium(III) {η5-C5Me4R} Complexes with Variable R Groups
Source: Int J Mol Sci. 2021 Jul 10;22(14):7422. doi: 10.3390/ijms22147422 (PMC8306930; doi:10.3390/ijms22147422)

checkCIF/PLATON report

Structure factors have been supplied for datablock(s) 2b, 3

THIS REPORT IS FOR GUIDANCE ONLY. IF USED AS PART OF A REVIEW PROCEDURE FOR PUBLICATION, IT SHOULD NOT REPLACE THE EXPERTISE OF AN EXPERIENCED CRYSTALLOGRAPHIC REFEREE.

No syntax errors found. CIF dictionary Interpreting this report

Datablock: 3

```
Bond precision:  C-C = 0.0046 A          Wavelength=0.71073

Cell:            a=13.9835(5)     b=8.8692(3)       c=15.3611(6)
                 alpha=90         beta=96.317(2)    gamma=90
Temperature:     293 K

                Calculated                 Reported
Volume          1893.55(12)                1893.55(12)
Space group     P 21/c                     P 21/c
Hall group      -P 2ybc                    -P 2ybc
Moiety formula  C17 H23 Cl2 Ir O2 S        ?
Sum formula     C17 H23 Cl2 Ir O2 S        C17 H23 Cl2 Ir O2 S
Mr              554.53                     554.51
Dx,g cm-3       1.945                      1.945
Z               4                          4
Mu (mm-1)       7.449                      7.449
F000            1072.0                     1072.0
F000'           1068.50
h,k,lmax        17,10,18                   17,10,18
Nref            3716                       3715
Tmin,Tmax       0.325,0.475                0.381,0.746
Tmin'           0.252

Correction method= # Reported T Limits: Tmin=0.381 Tmax=0.746
AbsCorr = EMPIRICAL

Data completeness= 1.000          Theta(max)= 25.992

R(reflections)= 0.0193( 3414)     wR2(reflections)= 0.0487( 3715)

S = 1.068                  Npar= 217
```

The following ALERTS were generated. Each ALERT has the format
test-name_ALERT_alert-type_alert-level.
Click on the hyperlinks for more details of the test.

Alert level C

```
PLAT480_ALERT_4_C Long H...A H-Bond Reported H1       ..S1       .       3.00 Ang.
PLAT911_ALERT_3_C Missing FCF Refl Between Thmin & STh/L=    0.600          2 Report
```

Alert level G

```
PLAT002_ALERT_2_G Number of Distance or Angle Restraints on AtSite          2 Note
PLAT172_ALERT_4_G The CIF-Embedded .res File Contains DFIX Records          1 Report
PLAT199_ALERT_1_G Reported _cell_measurement_temperature ..... (K)        293 Check
PLAT200_ALERT_1_G Reported   _diffrn_ambient_temperature ..... (K)        293 Check
PLAT860_ALERT_3_G Number of Least-Squares Restraints ............           1 Note
PLAT883_ALERT_1_G No Info/Value for _atom_sites_solution_primary .     Please Do !
PLAT913_ALERT_3_G Missing # of Very Strong Reflections in FCF ....          1 Note
PLAT933_ALERT_2_G Number of OMIT Records in Embedded .res File ...          2 Note
PLAT978_ALERT_2_G Number C-C Bonds with Positive Residual Density.          3 Info
```

0 **ALERT level A** = Most likely a serious problem - resolve or explain
0 **ALERT level B** = A potentially serious problem, consider carefully
2 **ALERT level C** = Check. Ensure it is not caused by an omission or oversight
9 **ALERT level G** = General information/check it is not something unexpected

3 ALERT type 1 CIF construction/syntax error, inconsistent or missing data
3 ALERT type 2 Indicator that the structure model may be wrong or deficient
3 ALERT type 3 Indicator that the structure quality may be low
2 ALERT type 4 Improvement, methodology, query or suggestion
0 ALERT type 5 Informative message, check

Datablock: 2b

Bond precision: C-C = 0.0087 A   Wavelength=0.71073

Cell:	a=14.4923(5)	b=7.3769(3)	c=15.5178(5)
	alpha=90	beta=95.905(1)	gamma=90
Temperature:	100 K		

	Calculated	Reported
Volume	1650.18(10)	1650.18(10)
Space group	P 21/n	P 21/n
Hall group	-P 2yn	-P 2yn
Moiety formula	C20 H21 Cl Ir N	?
Sum formula	C20 H21 Cl Ir N	C20 H21 Cl Ir N
Mr	503.05	503.03
Dx,g cm-3	2.025	2.025
Z	4	4
Mu (mm-1)	8.251	8.251
F000	968.0	968.0
F000’	963.38	
h,k,lmax	17,9,19	17,9,19
Nref	3236	3237
Tmin,Tmax	0.287,0.438	0.220,0.336
Tmin’	0.257	

Correction method= # Reported T Limits: Tmin=0.220 Tmax=0.336
AbsCorr = EMPIRICAL

Data completeness= 1.000 Theta(max)= 25.999

R(reflections)= 0.0275(3186) wR2(reflections)= 0.0702(3237)

S = 1.390 Npar= 213

The following ALERTS were generated. Each ALERT has the format
test-name_ALERT_alert-type_alert-level.
Click on the hyperlinks for more details of the test.

Alert level C

```
PLAT342_ALERT_3_C Low Bond Precision on  C-C Bonds ...............    0.00867 Ang.
PLAT906_ALERT_3_C Large K Value in the Analysis of Variance ......      2.643 Check
PLAT934_ALERT_3_C Number of (Iobs-Icalc)/Sigma(W) > 10 Outliers ..          1 Check
PLAT971_ALERT_2_C Check Calcd Resid. Dens.  1.61A   From C3              1.80 eA-3
PLAT971_ALERT_2_C Check Calcd Resid. Dens.  1.31A   From C12             1.74 eA-3
PLAT971_ALERT_2_C Check Calcd Resid. Dens.  0.92A   From Ir1             1.51 eA-3
PLAT972_ALERT_2_C Check Calcd Resid. Dens.  0.74A   From Ir1            -2.31 eA-3
PLAT972_ALERT_2_C Check Calcd Resid. Dens.  0.95A   From Ir1            -1.55 eA-3
PLAT977_ALERT_2_C Check Negative Difference Density on H11H             -0.35 eA-3
```

Alert level G

```
PLAT003_ALERT_2_G Number of Uiso or Uij Restrained non-H Atoms ...          6 Report
PLAT083_ALERT_2_G SHELXL Second Parameter in WGHT  Unusually Large      18.10 Why ?
PLAT168_ALERT_4_G The CIF-Embedded .res File Contains EXYZ Records          2 Report
PLAT171_ALERT_4_G The CIF-Embedded .res File Contains EADP Records          2 Report
PLAT186_ALERT_4_G The CIF-Embedded .res File Contains ISOR Records          1 Report
PLAT301_ALERT_3_G Main Residue  Disorder ..............(Resd  1  )         9% Note
PLAT380_ALERT_4_G Incorrectly? Oriented X(sp2)-Methyl Moiety .....       C113 Check
PLAT860_ALERT_3_G Number of Least-Squares Restraints .............         36 Note
PLAT883_ALERT_1_G No Info/Value for _atom_sites_solution_primary .     Please Do !
PLAT978_ALERT_2_G Number C-C Bonds with Positive Residual Density.          1 Info
```

---

0 **ALERT level A** = Most likely a serious problem - resolve or explain
0 **ALERT level B** = A potentially serious problem, consider carefully
9 **ALERT level C** = Check. Ensure it is not caused by an omission or oversight
10 **ALERT level G** = General information/check it is not something unexpected

1 ALERT type 1 CIF construction/syntax error, inconsistent or missing data
9 ALERT type 2 Indicator that the structure model may be wrong or deficient
5 ALERT type 3 Indicator that the structure quality may be low
4 ALERT type 4 Improvement, methodology, query or suggestion
0 ALERT type 5 Informative message, check

It is advisable to attempt to resolve as many as possible of the alerts in all categories. Often the minor alerts point to easily fixed oversights, errors and omissions in your CIF or refinement strategy, so attention to these fine details can be worthwhile. In order to resolve some of the more serious problems it may be necessary to carry out additional measurements or structure refinements. However, the purpose of your study may justify the reported deviations and the more serious of these should normally be commented upon in the discussion or experimental section of a paper or in the "special_details" fields of the CIF. checkCIF was carefully designed to identify outliers and unusual parameters, but every test has its limitations and alerts that are not important in a particular case may appear. Conversely, the absence of alerts does not guarantee there are no aspects of the results needing attention. It is up to the individual to critically assess their own results and, if necessary, seek expert advice.

Publication of your CIF in IUCr journals

A basic structural check has been run on your CIF. These basic checks will be run on all CIFs submitted for publication in IUCr journals (*Acta Crystallographica*, *Journal of Applied Crystallography*, *Journal of Synchrotron Radiation*); however, if you intend to submit to *Acta Crystallographica Section C* or *E* or *IUCrData*, you should make sure that full publication checks are run on the final version of your CIF prior to submission.

Publication of your CIF in other journals

Please refer to the *Notes for Authors* of the relevant journal for any special instructions relating to CIF submission.

PLATON version of 16/05/2021; check.def file version of 13/05/2021

Datablock 3 - ellipsoid plot

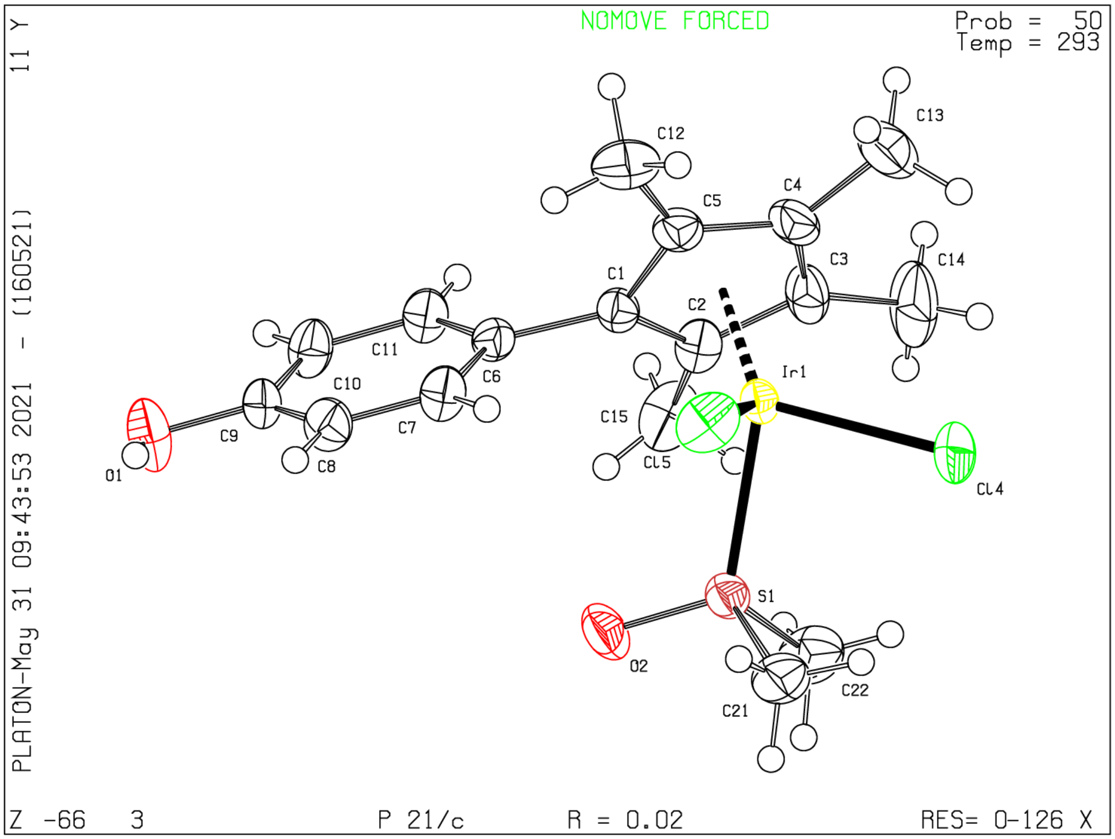

Datablock 2b - ellipsoid plot

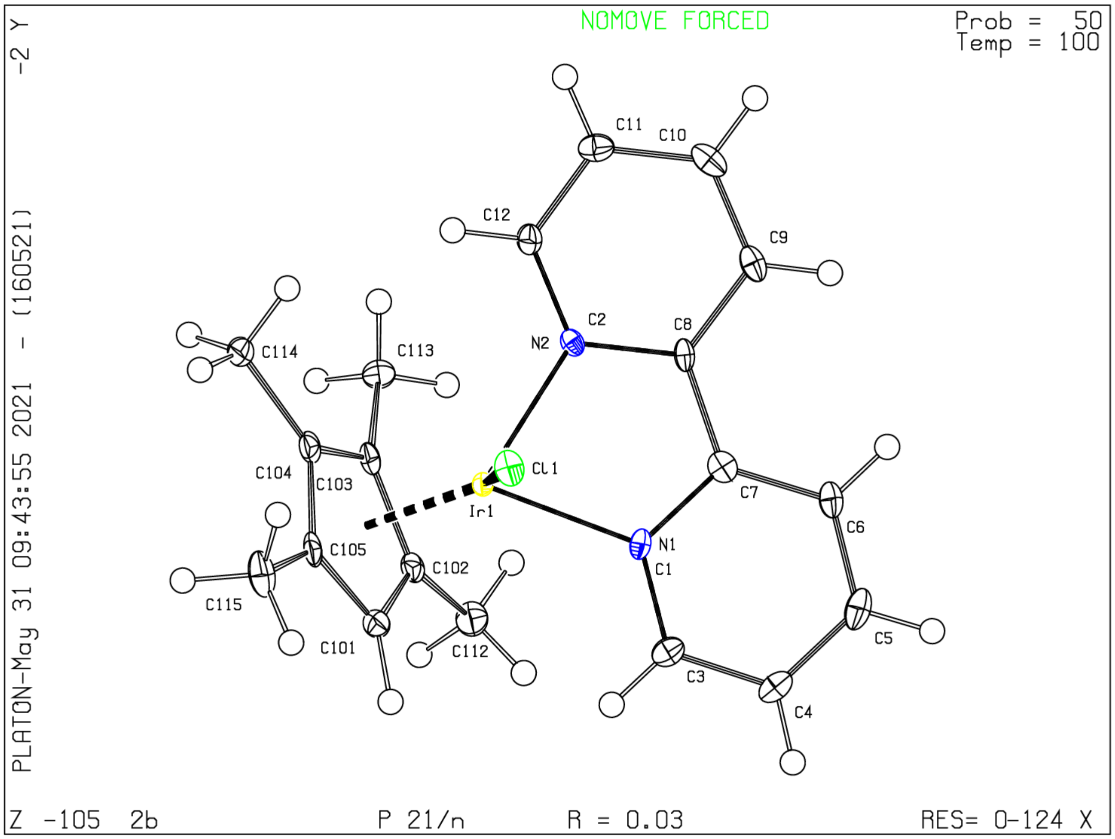

Supplement: Supplementary file 1 [file ijms-22-07422-s001.zip › Supplementary Files/checkcif.pdf]
