# Supplementary material for: A Comparative Analysis of the In Vitro Anticancer Activity of Iridium(III) {η5-C5Me4R} Complexes with Variable R Groups
Source: Int J Mol Sci. 2021 Jul 10;22(14):7422. doi: 10.3390/ijms22147422 (PMC8306930; doi:10.3390/ijms22147422)

A Comparative Analysis of the In Vitro Anticancer Activity of Iridium(III) {η^5-C_5Me_4R} Complexes with Variable R Groups

Alice De Palo,[a] Dijana Draca,[b] Maria Grazia Murrali,[a] Stefano Zacchini,[c] Guido Pampaloni,[a] Sanja Mijatovic,[b] Danijela Maksimovic-Ivanic,[b,*] Fabio Marchetti [a,*]

[a] University of Pisa, Dipartimento di Chimica e Chimica Industriale, Via Moruzzi 13, I-56124 Pisa, Italy.

[b] University of Belgrade, Department of Immunology, Institute for Biological Research "Sinisa Stankovic" - National Institute of Republic of Serbia, 11060 Belgrade, Serbia.

[c] University of Bologna, Dipartimento di Chimica Industriale "Toso Montanari", Viale Risorgimento 4, I-40136 Bologna, Italy.

Supporting Information

<u>**Table of contents**</u> *Pages*

Figures S1-S15: NMR spectra of complexes S2-S10

Figures S16: Effect of **1b,c-e** on viability of A2780 cells S11

Figures S17: Effect of **2a-d** and **3** on viability of A2780 cells S12

Figures S18: Effect of **2b** and **2d** on viability of macrophages S13

Figures S19: Autophagosomes presence in A2780 cells treated with **2b** and **2d** S14

Figure S1. ^{1}H NMR spectrum (401 MHz, $CDCl_3$) of **2b**

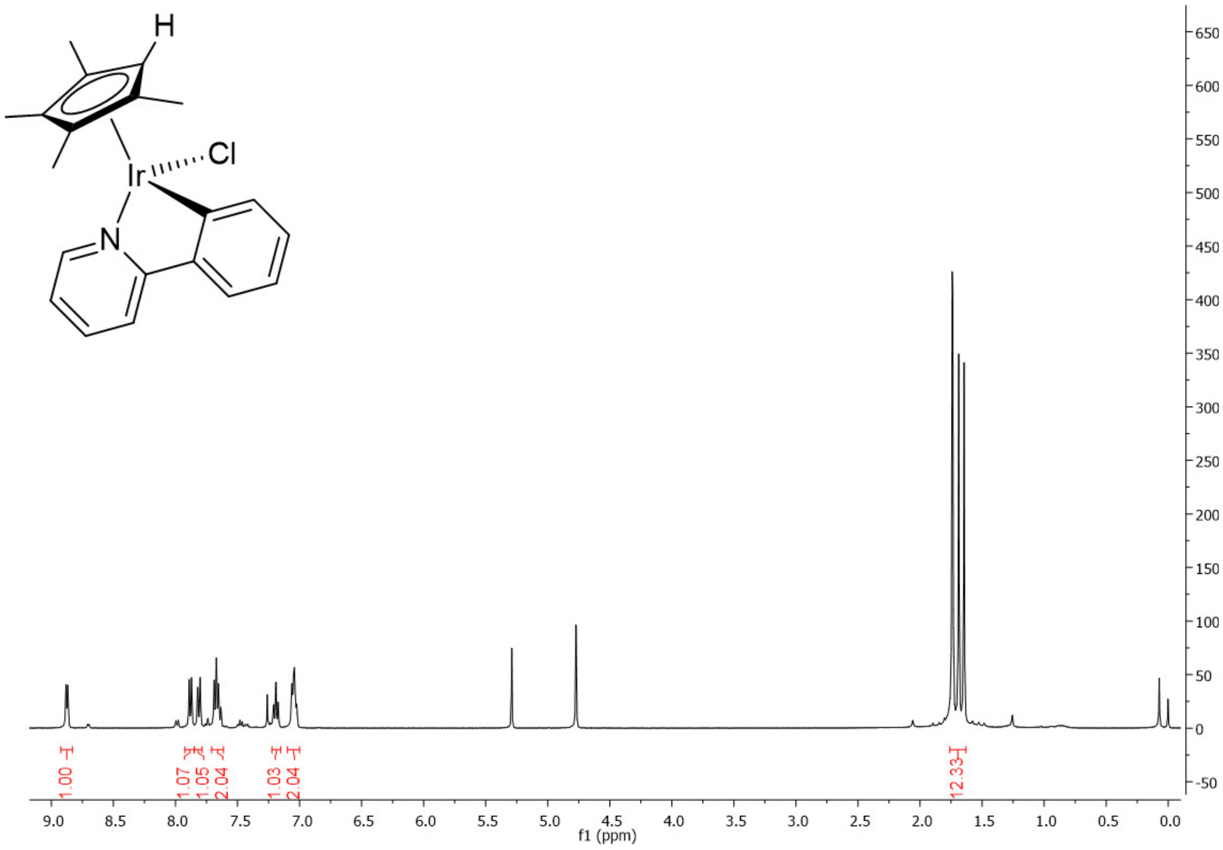

Figure S2. $^{13}C\{^{1}H\}$ NMR spectrum (101 MHz, $CDCl_3$) of **2b**.

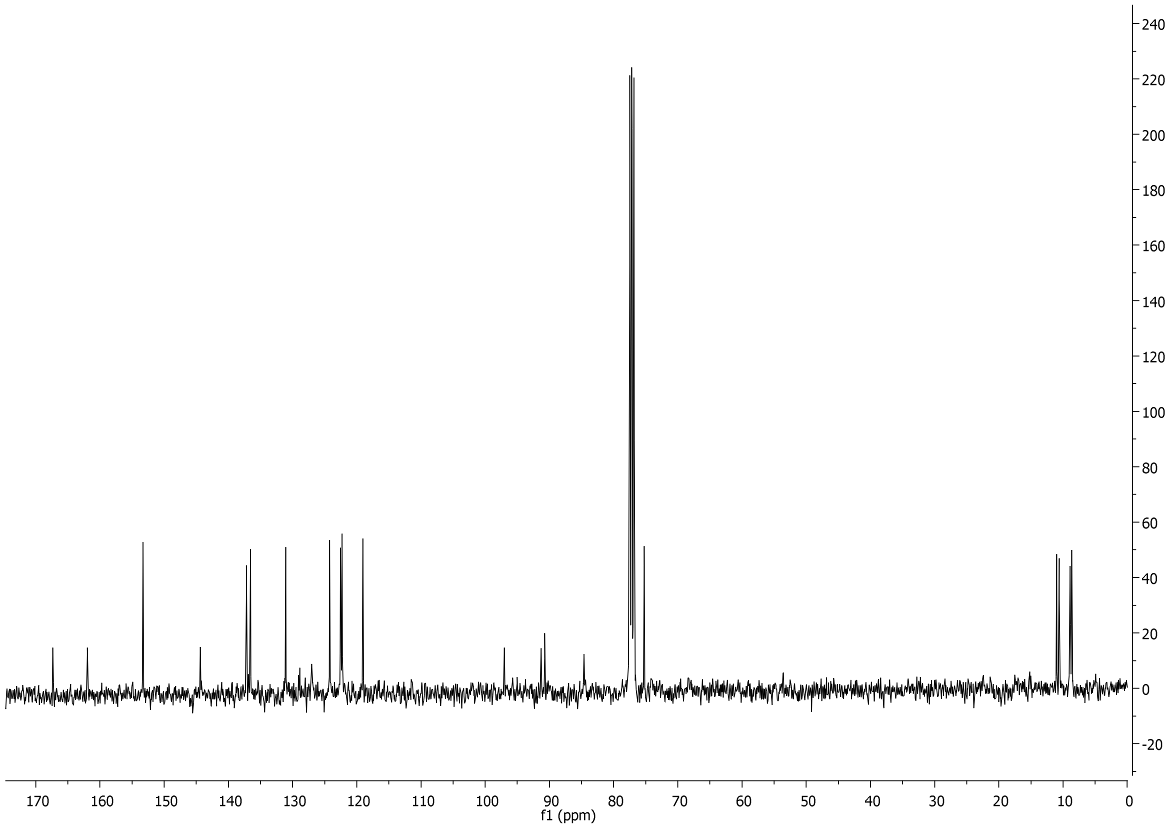

Figure S3. ^{1}**H NMR spectrum (401 MHz,** $CDCl_3$**) of 2c.**

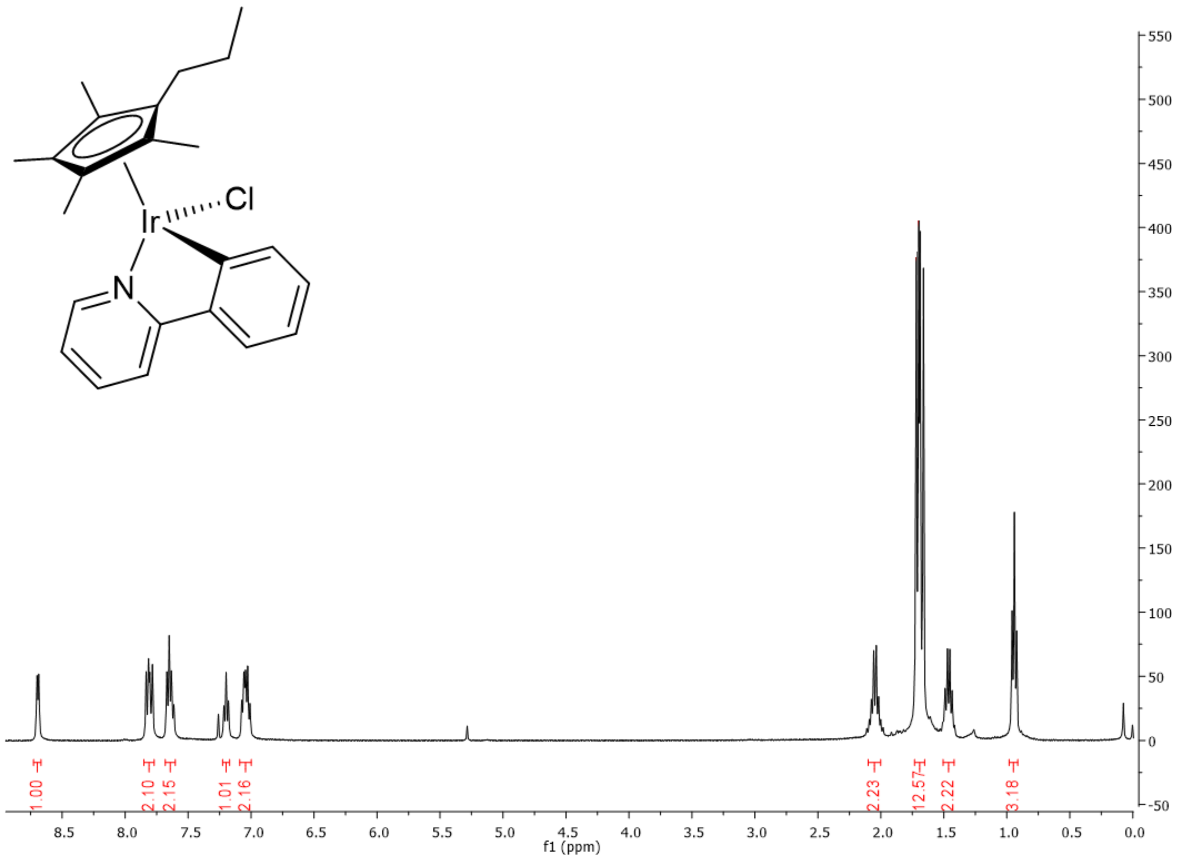

Figure S4. $^{13}C\{^{1}H\}$ **NMR spectrum (101 MHz,** $CDCl_3$**) of 2c.**

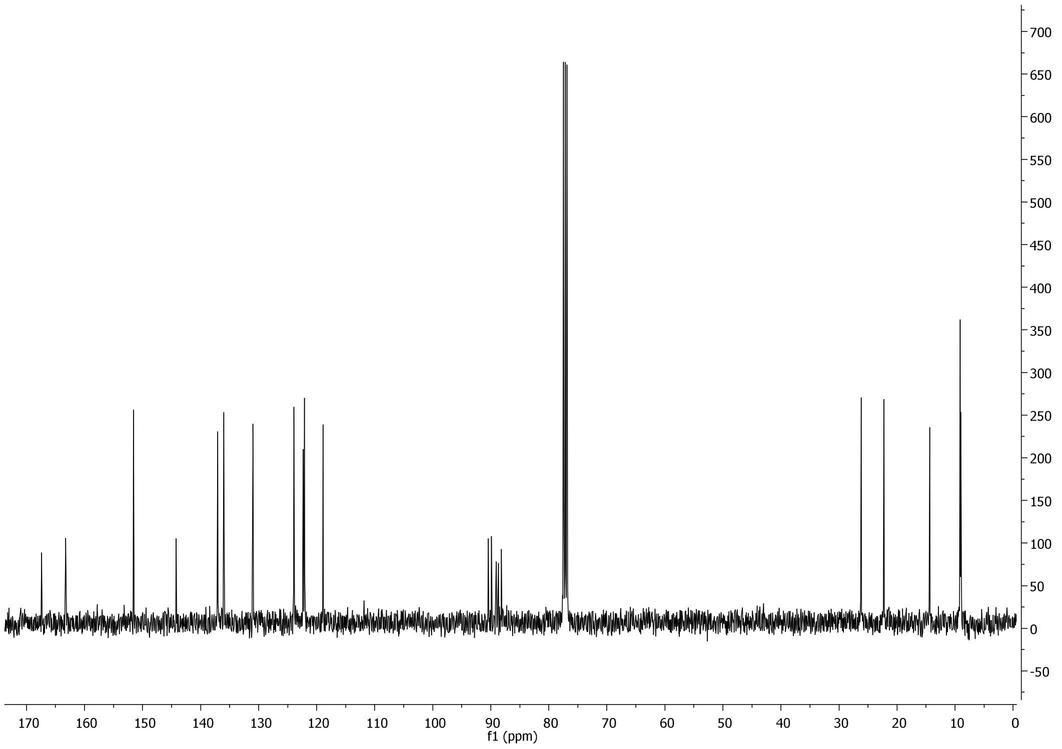

Figure S5. ^{1}**H NMR spectrum (401 MHz,** $CDCl_3$**) of 2d.**

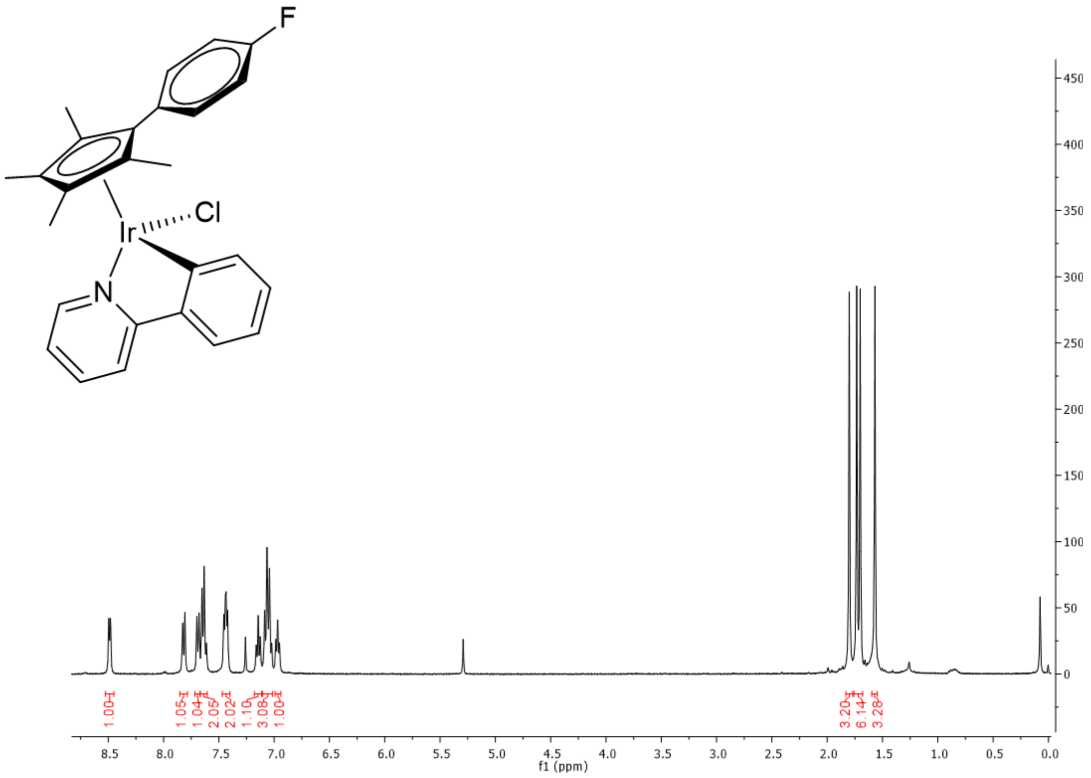

Figure S6. $^{13}C\{^1H\}$ **NMR spectrum (101 MHz,** $CDCl_3$**) of 2d.**

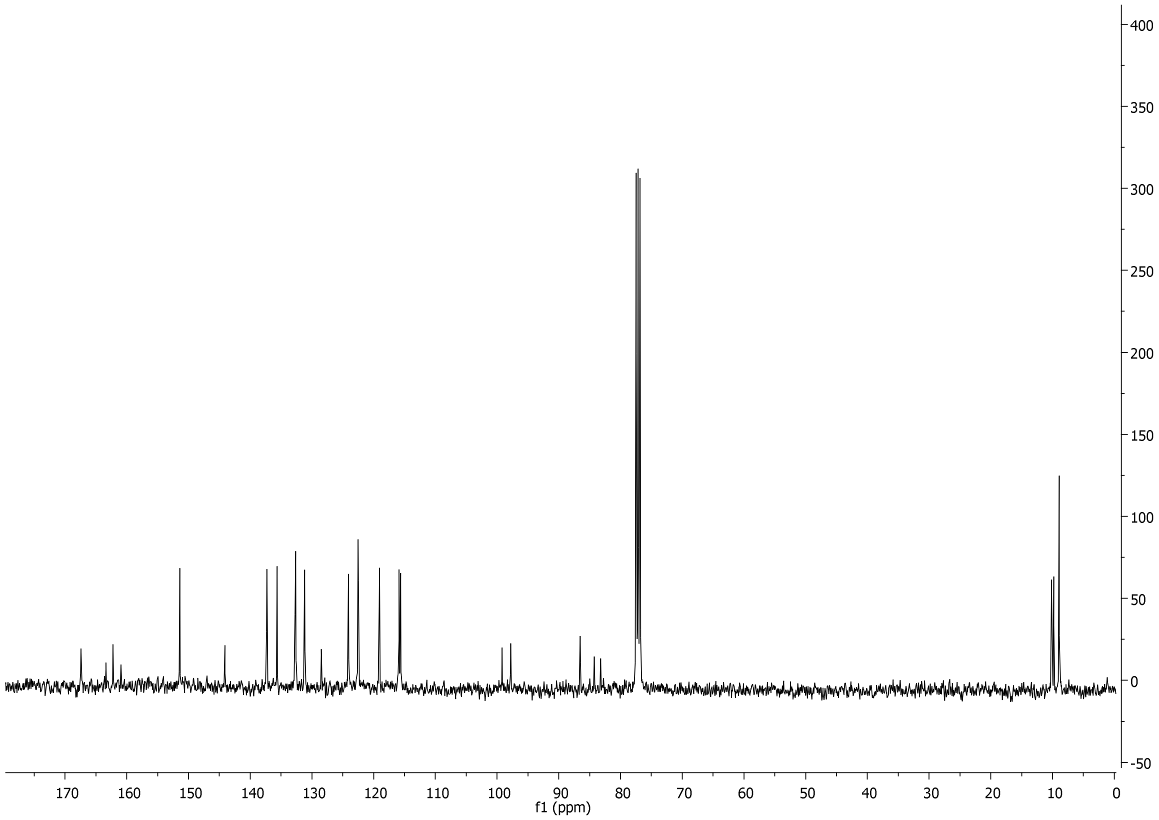

Figure S7. $^{19}F\{^1H\}$ NMR spectrum (378 MHz, $CDCl_3$) of 2d. Inset shows the -110 / -120 ppm region.

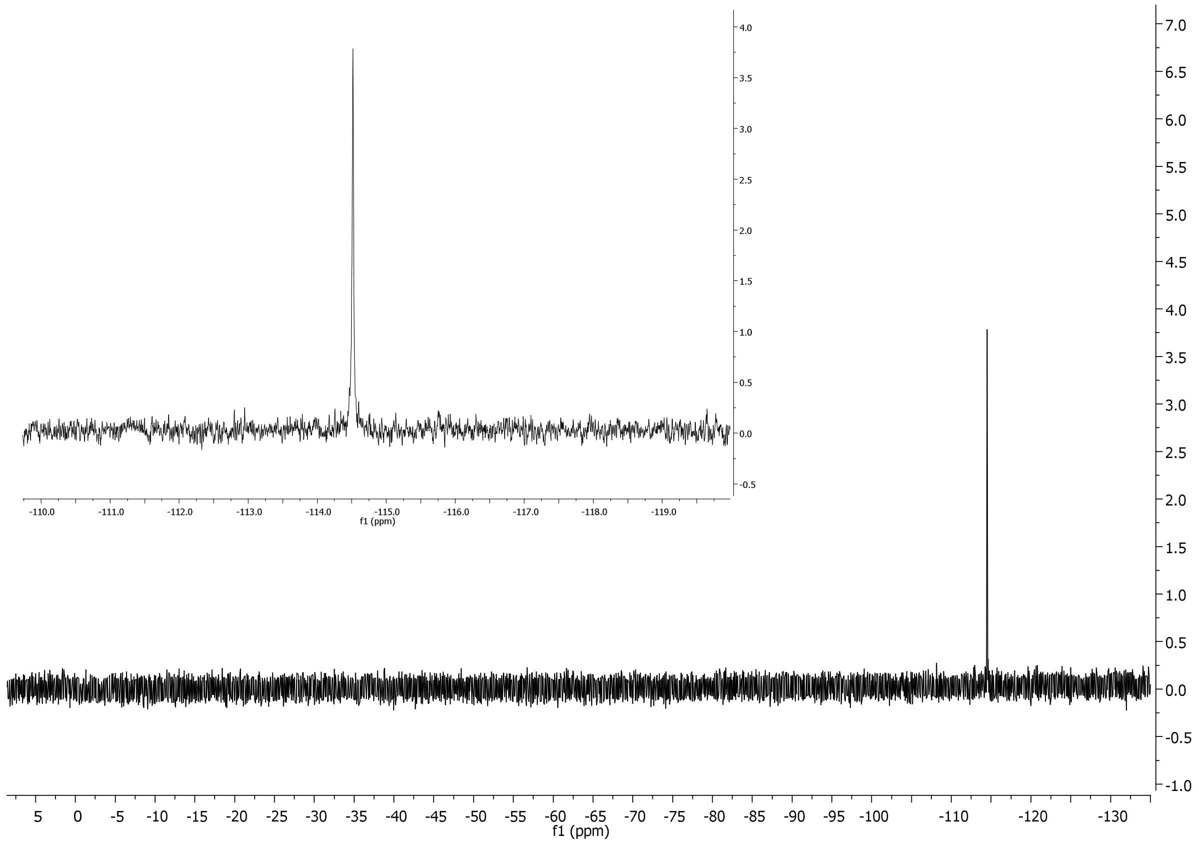

Figure S8. ^{1}H NMR spectrum (401 MHz, $CDCl_3$) of 3.

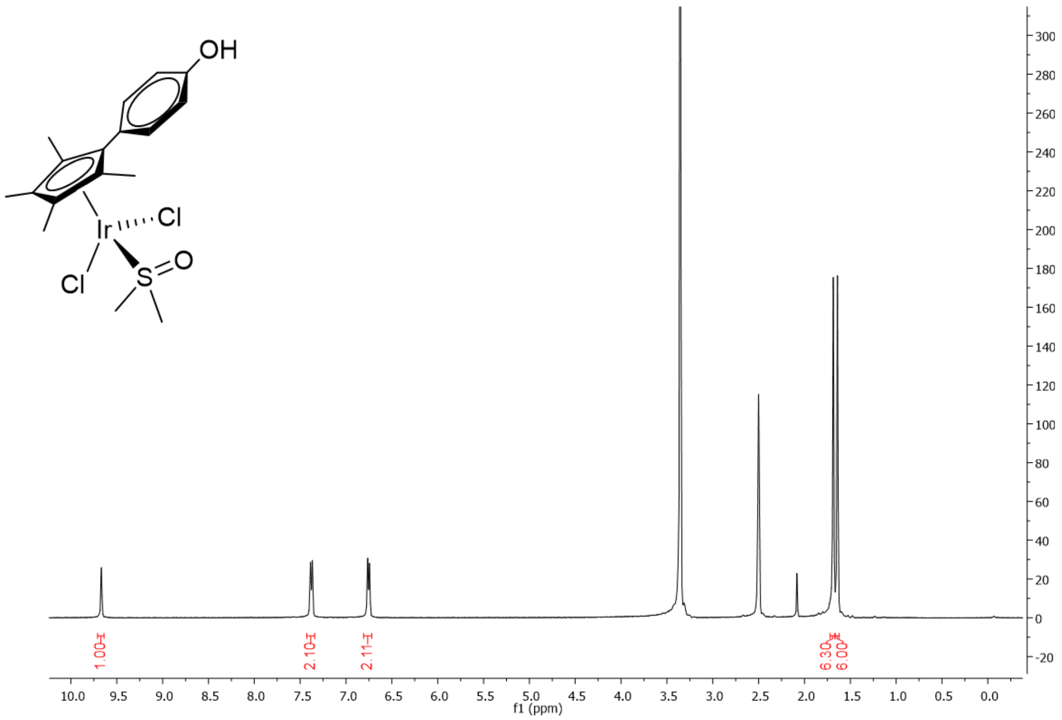

Figure S9. $^{13}C\{^1H\}$ NMR spectrum (101 MHz, $CDCl_3$) of 3.

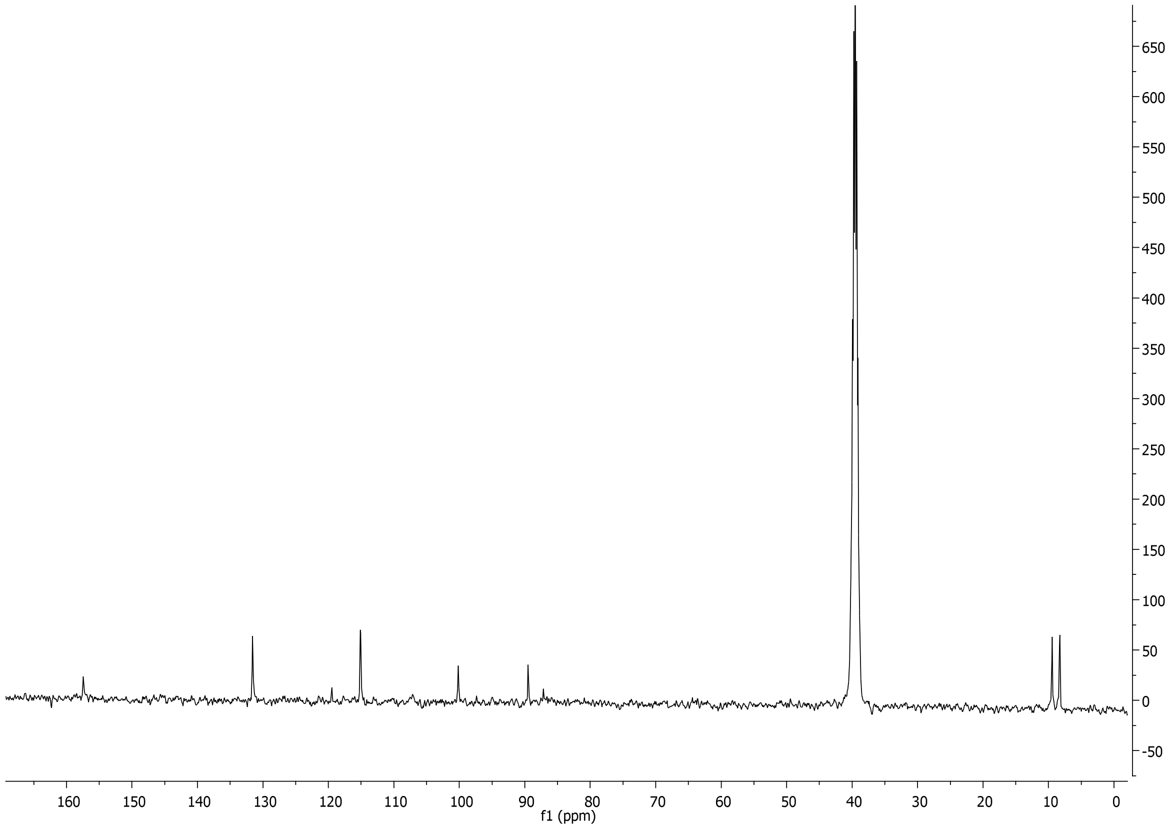

Figure S10. ^{1}H NMR spectrum (401 MHz, dmso-d_6/D_2O) of **2a**.

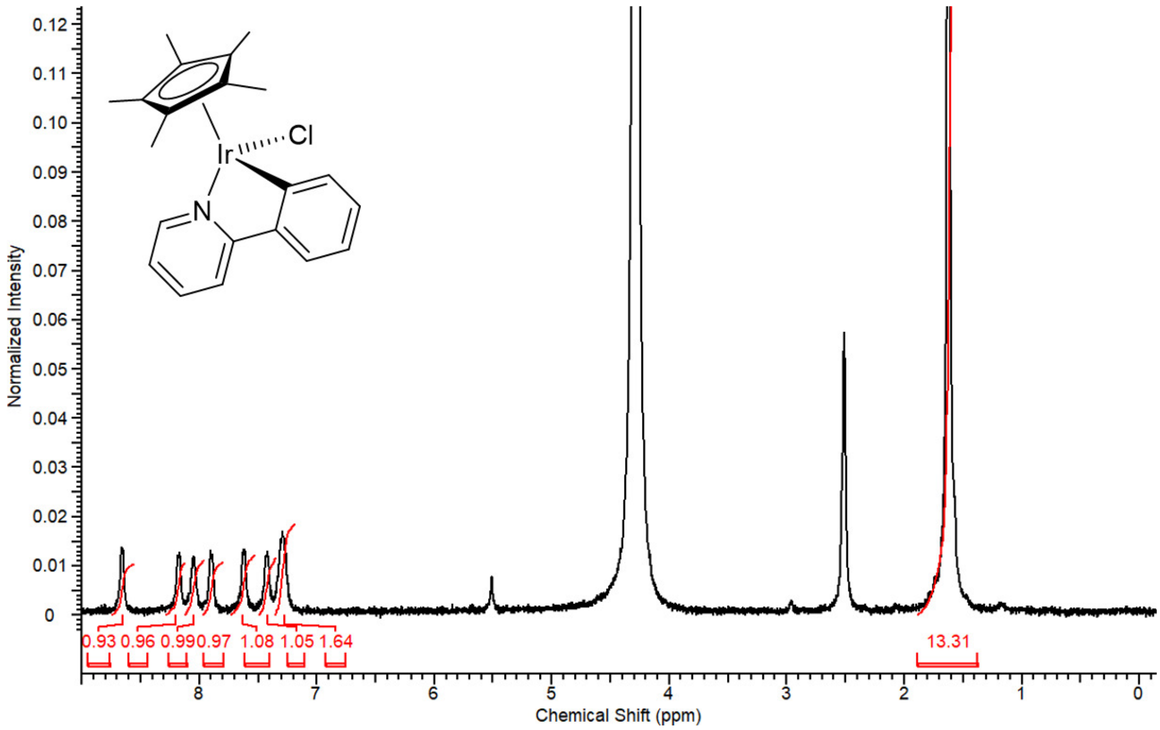

Figure S11. ^{1}H NMR spectrum (401 MHz, dmso-d_6/D_2O) of **2b**.

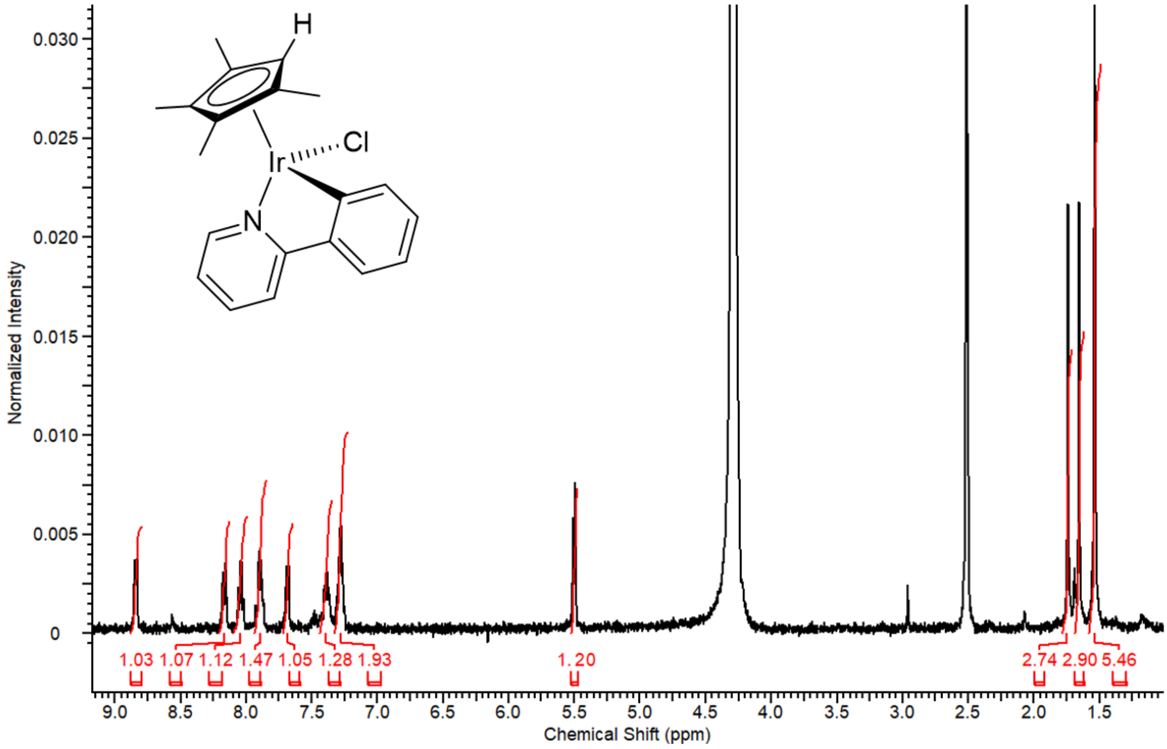

Figure S12. ^{1}H **NMR spectrum (401 MHz, dmso-d$_6$/D$_2$O) of 2c.**

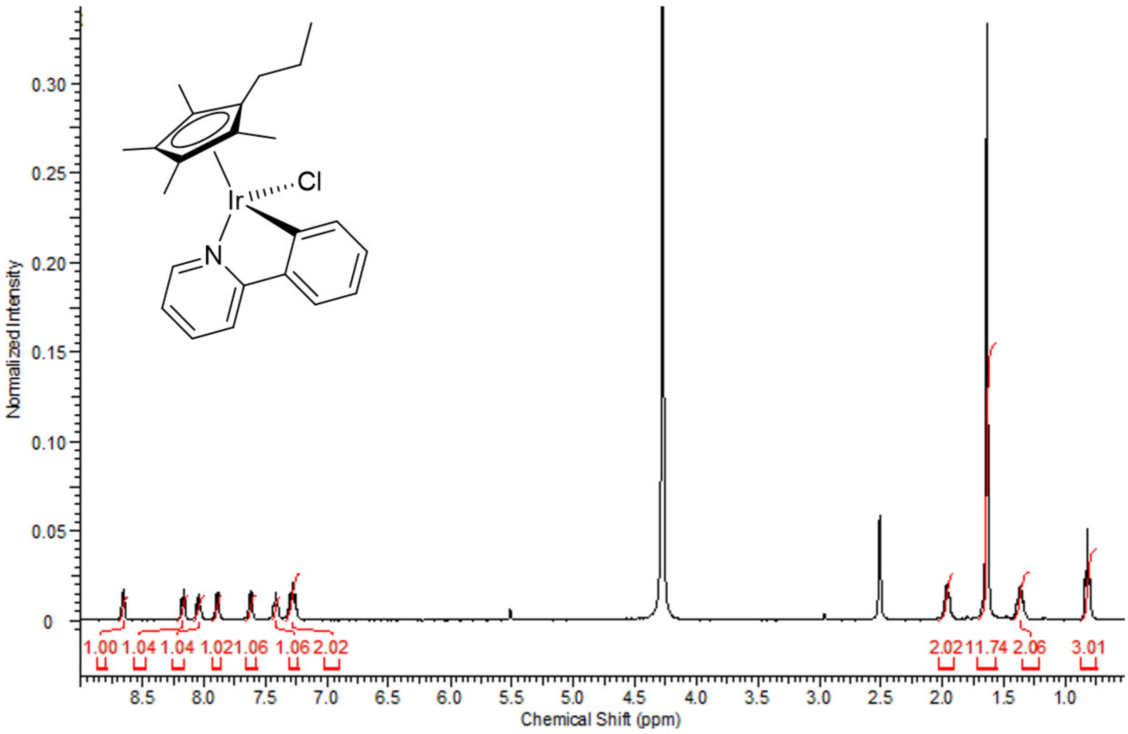

Figure S13. ^{1}H **NMR spectrum (401 MHz, dmso-d$_6$/D$_2$O) of 2d.**

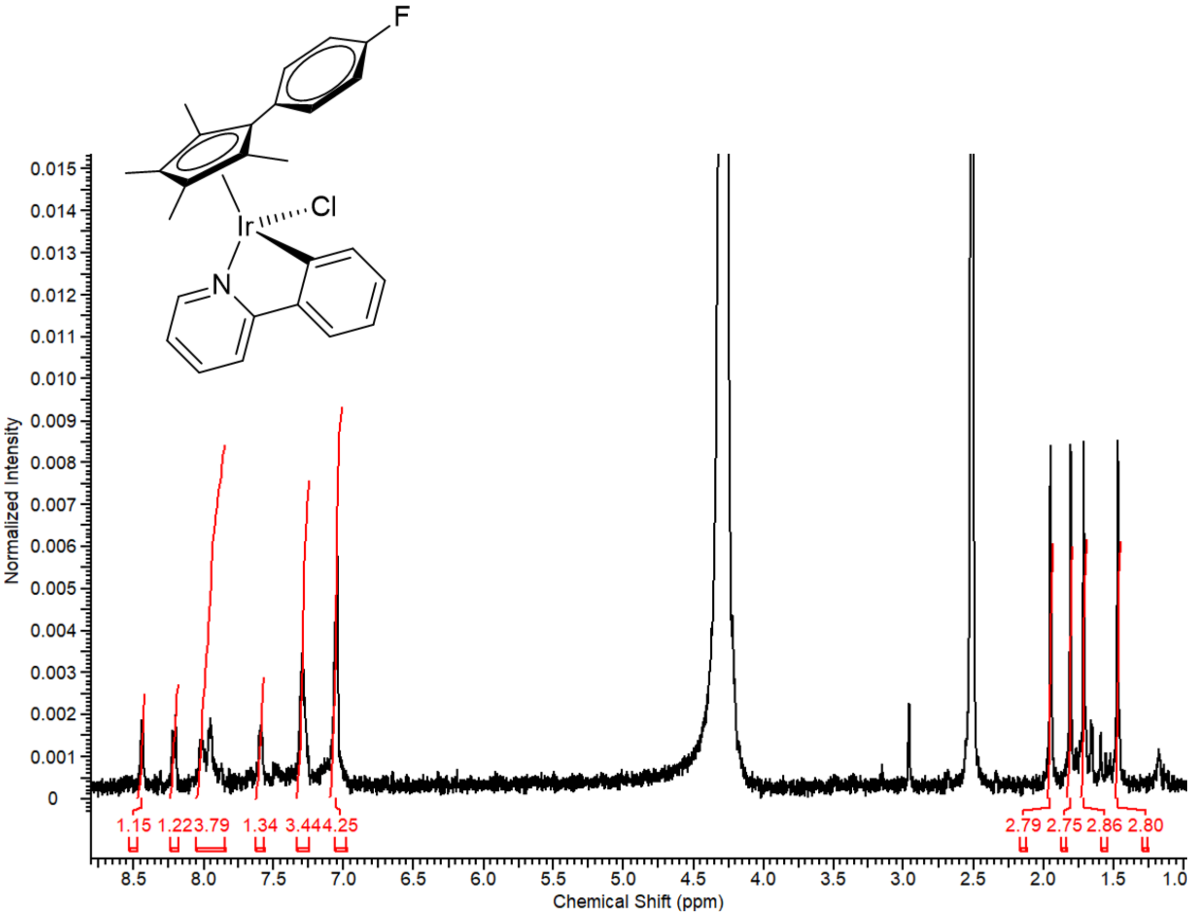

Figure S14. $^{19}F\{^{1}H\}$ **NMR spectrum (378 MHz, dmso-d_6/D_2O) of 2d.**

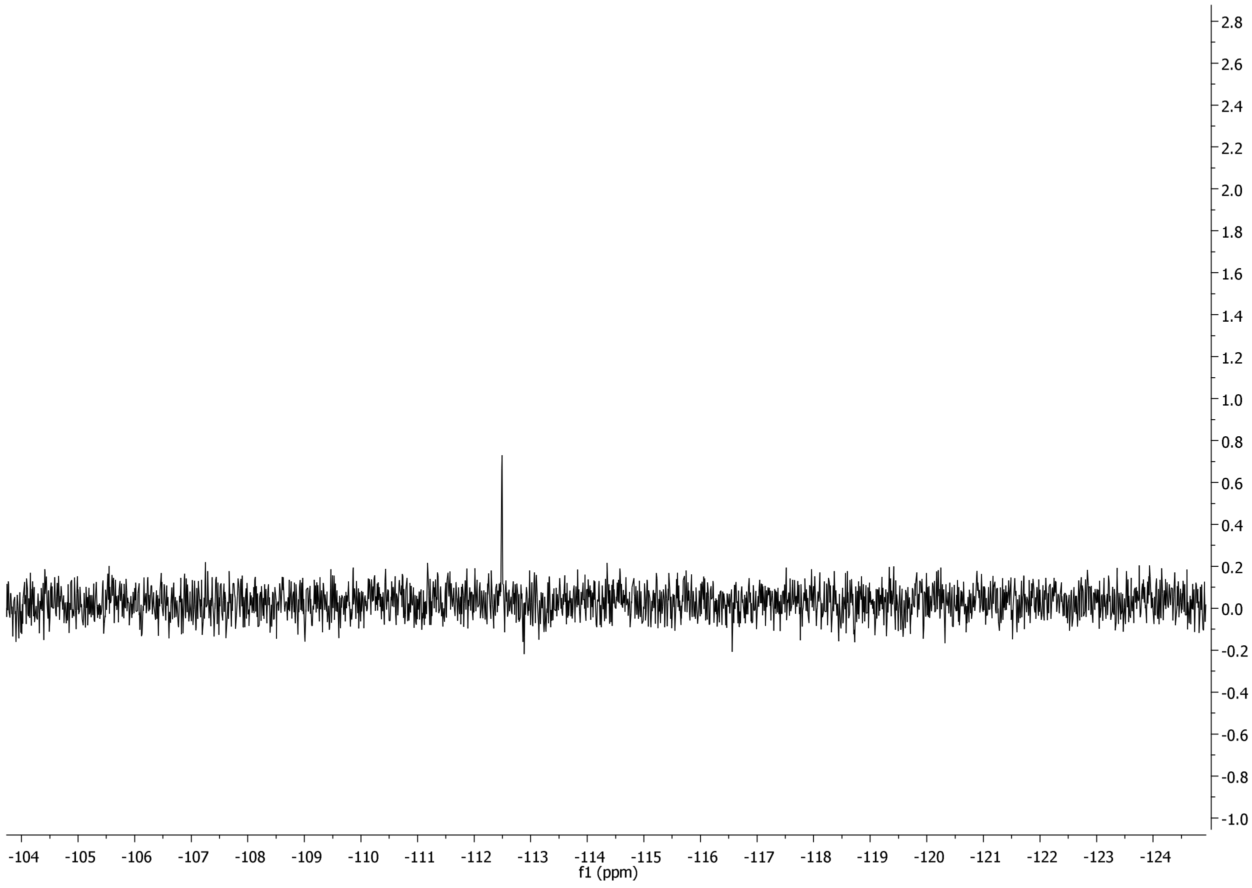

Figure S15. **^{1}H NMR spectrum (401 MHz, dmso-d_6/D_2O) of 3.**

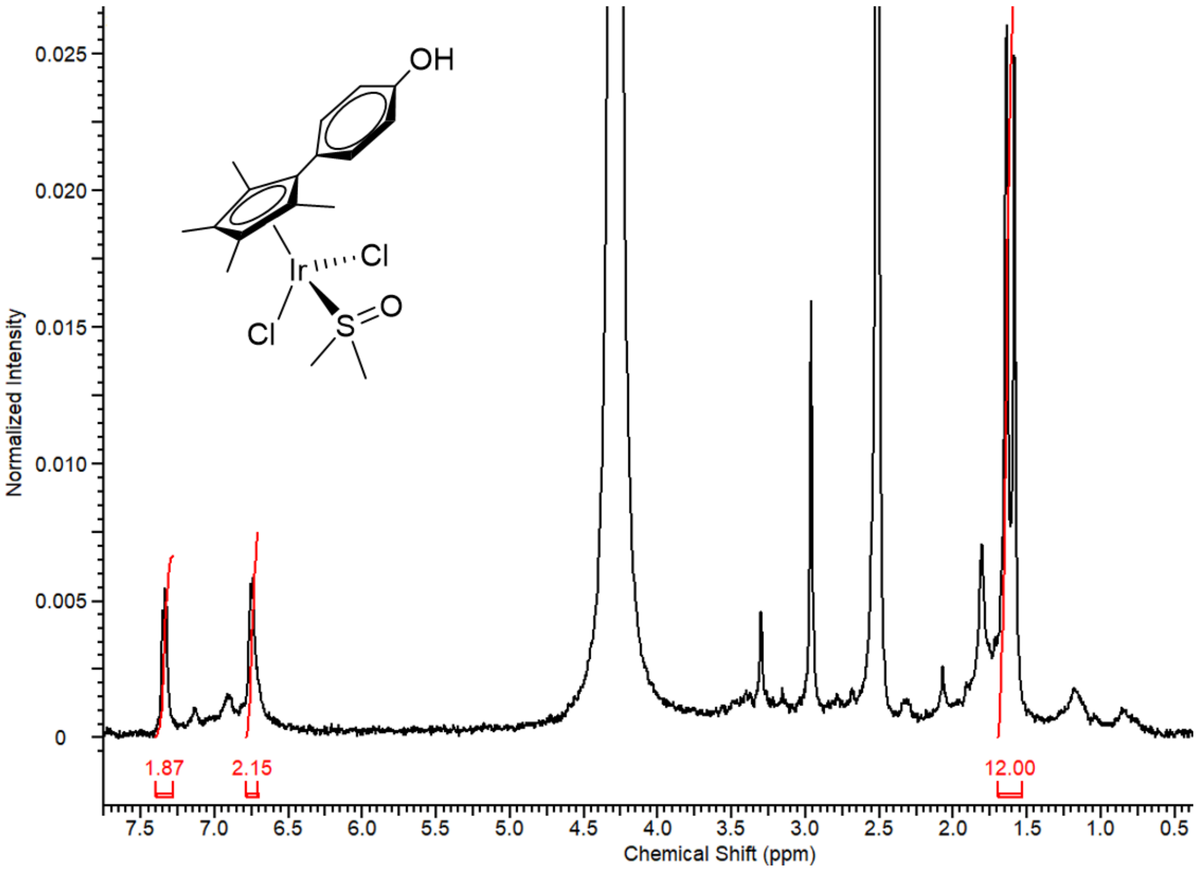

Figure S16. Effect of **1b,c-e** on viability of A2780 cells (left panel – MTT assay, right panel – CV assay; * p < 0.05 refers to untreated culture).

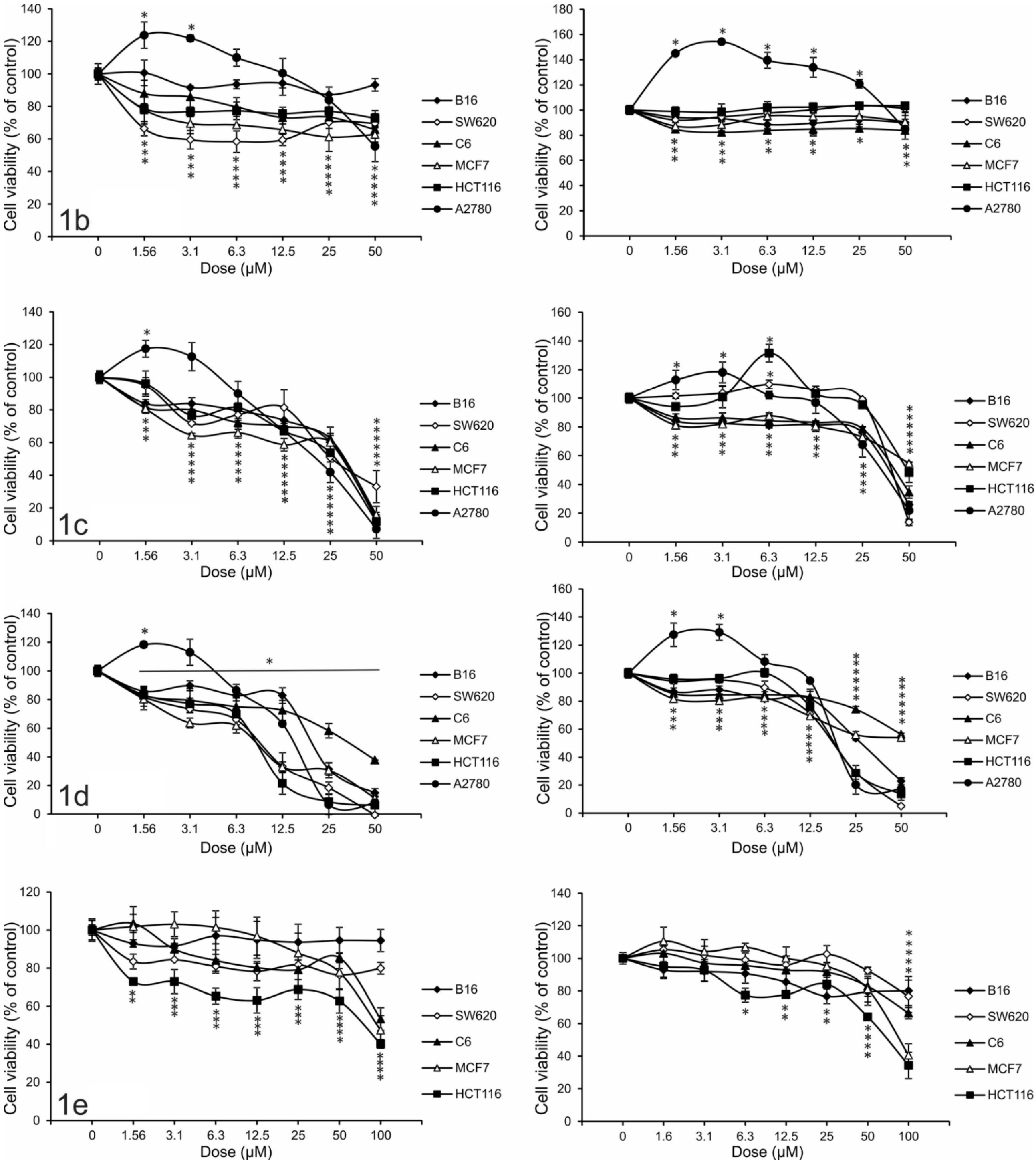

Figure S17. Effect of **2a-d** and **3** on viability of A2780 cells (left panel – MTT assay, right panel – CV assay; * $p < 0.05$ refers to untreated cultures).

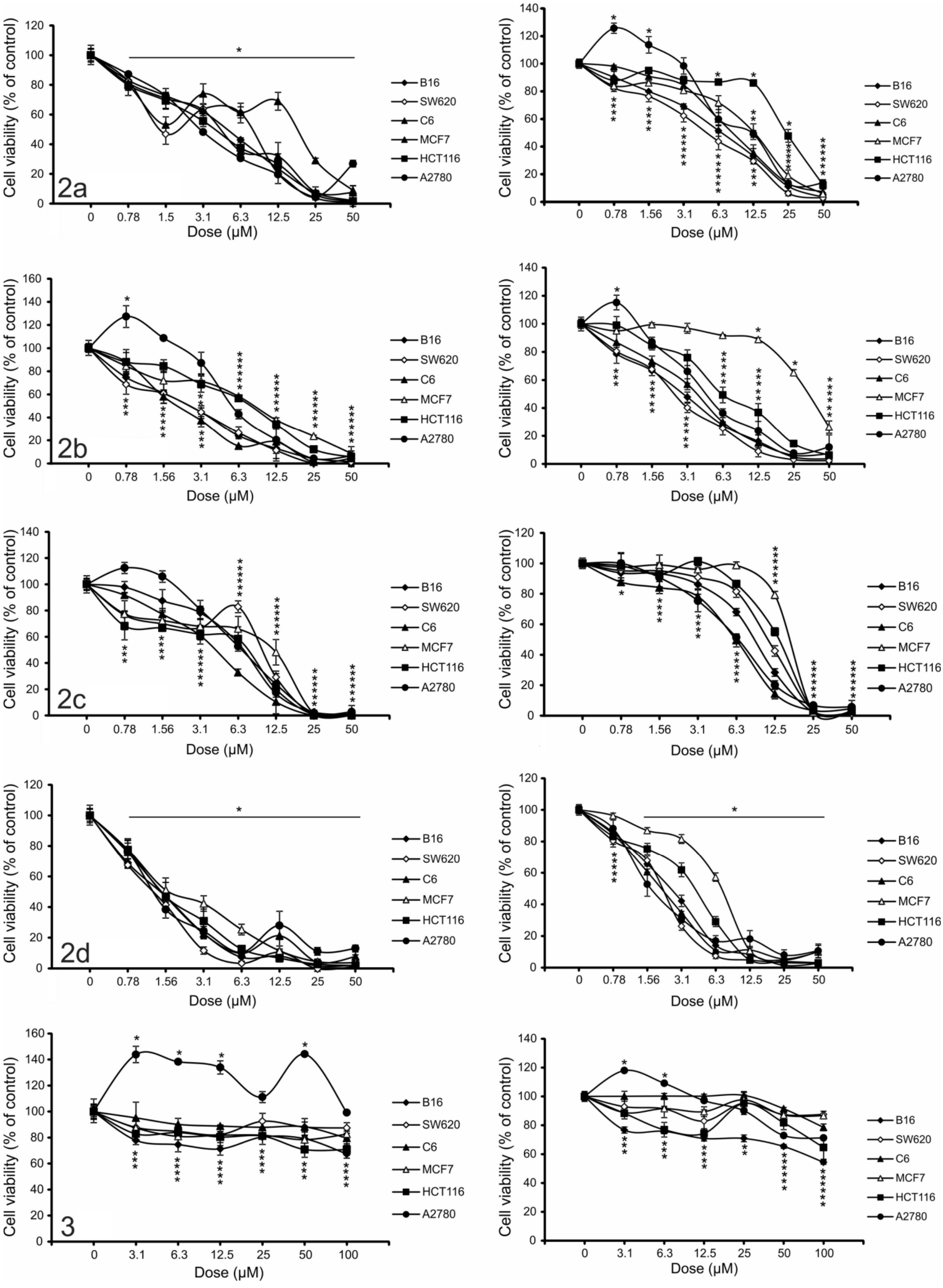

Figure S18. Effect of **2b** and **2d** on viability of macrophages (CV assay; * $p < 0.05$ refers to untreated culture).

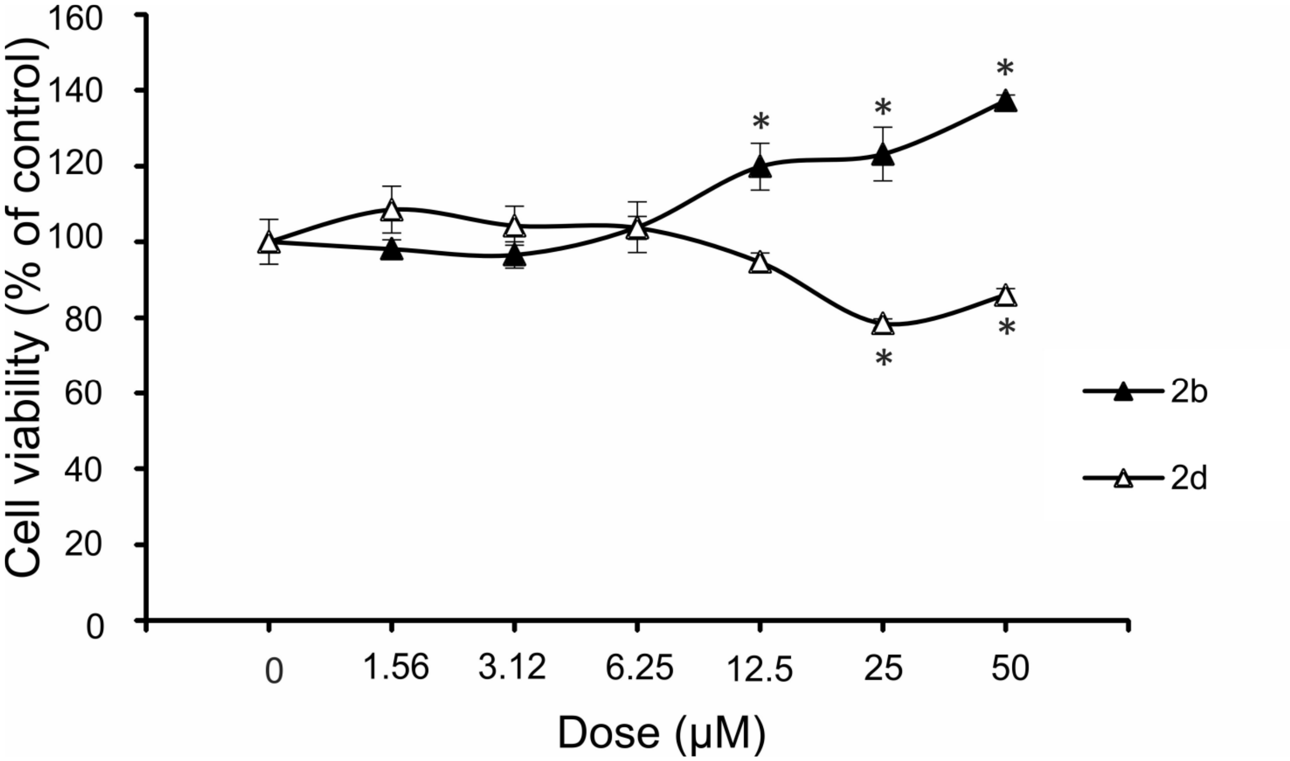

Figure S19. Autophagosomes presence (AO assay) in A2780 cells treated with **2b** and **2d**.

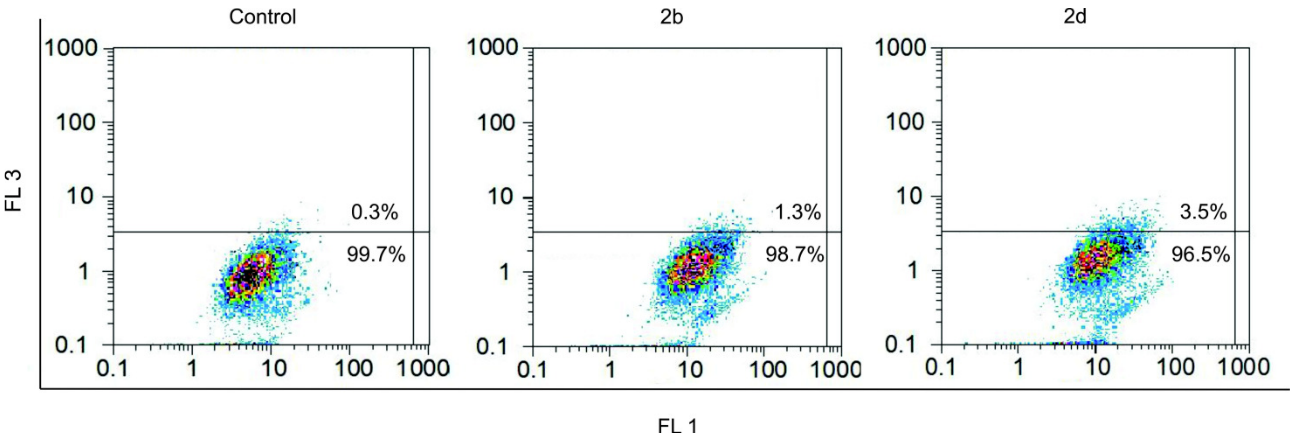

Supplement: Supplementary file 1 [file ijms-22-07422-s001.zip › Supplementary Files/Supporting Information.pdf]
